# A one-pot synthesis of *N*^2^,6-diaryl-5,6-dihydro-1,3,5-triazine-2,4-diamines and systematic evaluation of their ability to host ethanol in crystals†

**DOI:** 10.1039/c9ra08795h

**Published:** 2019-11-19

**Authors:** Ahmad Junaid, Yee Seng Tan, Edward R. T. Tiekink, Anton V. Dolzhenko

**Affiliations:** School of Pharmacy, Monash University Malaysia Jalan Lagoon Selatan Bandar Sunway Selangor Darul Ehsan 47500 Malaysia anton.dolzhenko@monash.edu; Research Centre for Crystalline Materials, School of Science and Technology, 5 Jalan Universiti, Sunway University Bandar Sunway Selangor Darul Ehsan 47500 Malaysia; School of Pharmacy and Biomedical Sciences, Curtin Health Innovation Research Institute, Faculty of Health Sciences, Curtin University GPO Box U1987 Perth Western Australia 6845 Australia; Department of Chemistry, Prairie View A&M University P.O. Box 519 Prairie View Texas 77446 USA

## Abstract

A convenient one-pot method for the preparation of *N*^2^,6-diaryl-5,6-dihydro-1,3,5-triazine-2,4-diamines was developed using a three-component synthesis of 1,6-diaryl-1,6-dihydro-1,3,5-triazine-2,4-diamines followed by their Dimroth rearrangement to the desired products. The prepared compounds crystallized from ethanol as ethanol clathrates (1 : 1). X-ray crystallography on several products confirmed the adoption of 5,6-dihydro-tautomer. The thermal analysis and powder X-ray diffraction experiments on selected compounds suggested that thermal desolvation of crystals was irreversible.

## Abstract 2

One-pot synthesis and analysis of stable dihydrotriazine-EtOH clathrates.

## Graphical abstract

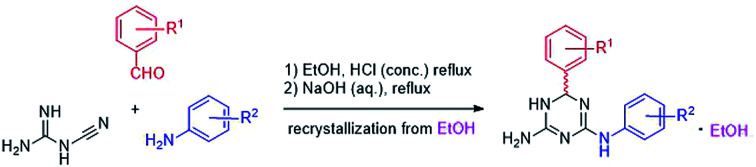

## Introduction

**Scheme 1:**
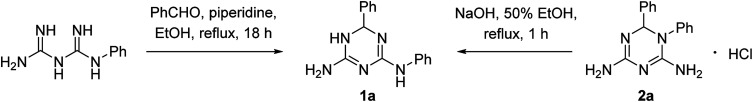
Reported^1^ syntheses of *N*^2^,6-diphenyl-5,6-dihydro-1,3,5-triazine-2,4-diamine (1a).

The first synthesis of *N*^2^,6-diphenyl-5,6-dihydro-1,3,5-triazine-2,4-diamine (1a) was reported in 1956 by Modest and Levine.^1^ They prepared 1a using a piperidine-catalyzed condensation of *N*-phenylbiguanide with benzaldehyde and alternatively, by a Dimroth rearrangement of 1,6-diphenyl-1,6-dihydro-1,3,5-triazine-2,4-diamine hydrochloride (2a) upon its heating with a base (Scheme 1).

Despite a significant interest in 1,3,5-triazine derivatives as bioactive compounds, particularly due to their anticancer properties,^2^ since that time no reports on *N*^2^,6-diphenyl-5,6-dihydro-1,3,5-triazine-2,4-diamine (1a) and its analogues had appeared until recently. In 2019, a Japanese patent^3^ mentioned another *N*^2^,6-diaryl-5,6-dihydro-1,3,5-triazine-2,4-diamine, compound 1b, as an agent against esophageal squamous cell carcinoma. However, the preparation of this compound was not described. With our interest towards development of new practical methods for the multicomponent synthesis of bioactive 1,3,5-triazines,^4,5^ we report herein a one-pot synthesis of *N*^2^,6-diaryl-5,6-dihydro-1,3,5-triazine-2,4-diamines, including 1a and 1b. The yield of 1a in the rearrangement of 2a described by Modest and Levine^1^ was not reported, but the fact that 2a could be effectively prepared *via* a three-component reaction of cyanoguanidine, aniline and benzaldehyde^6^ prompted us to combine these two reactions in a one-pot procedure.
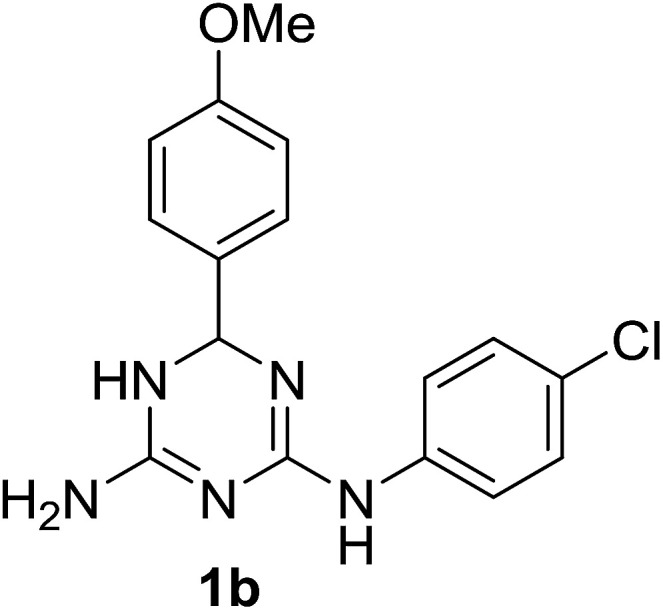


Modest and Levine reported^1^ that recrystallization of *N*^2^,6-diphenyl-5,6-dihydro-1,3,5-triazine-2,4-diamine (1a) from ethanol resulted in the formation of an ethanol solvate (1 : 1). In our study, we attempted to explore whether this ability to host ethanol is common for analogues of 1a substituted at the phenyl rings. We also solved crystal structures of several of these ethanol clathrates and assessed their stability.

## Results and discussion

**Scheme 2:**
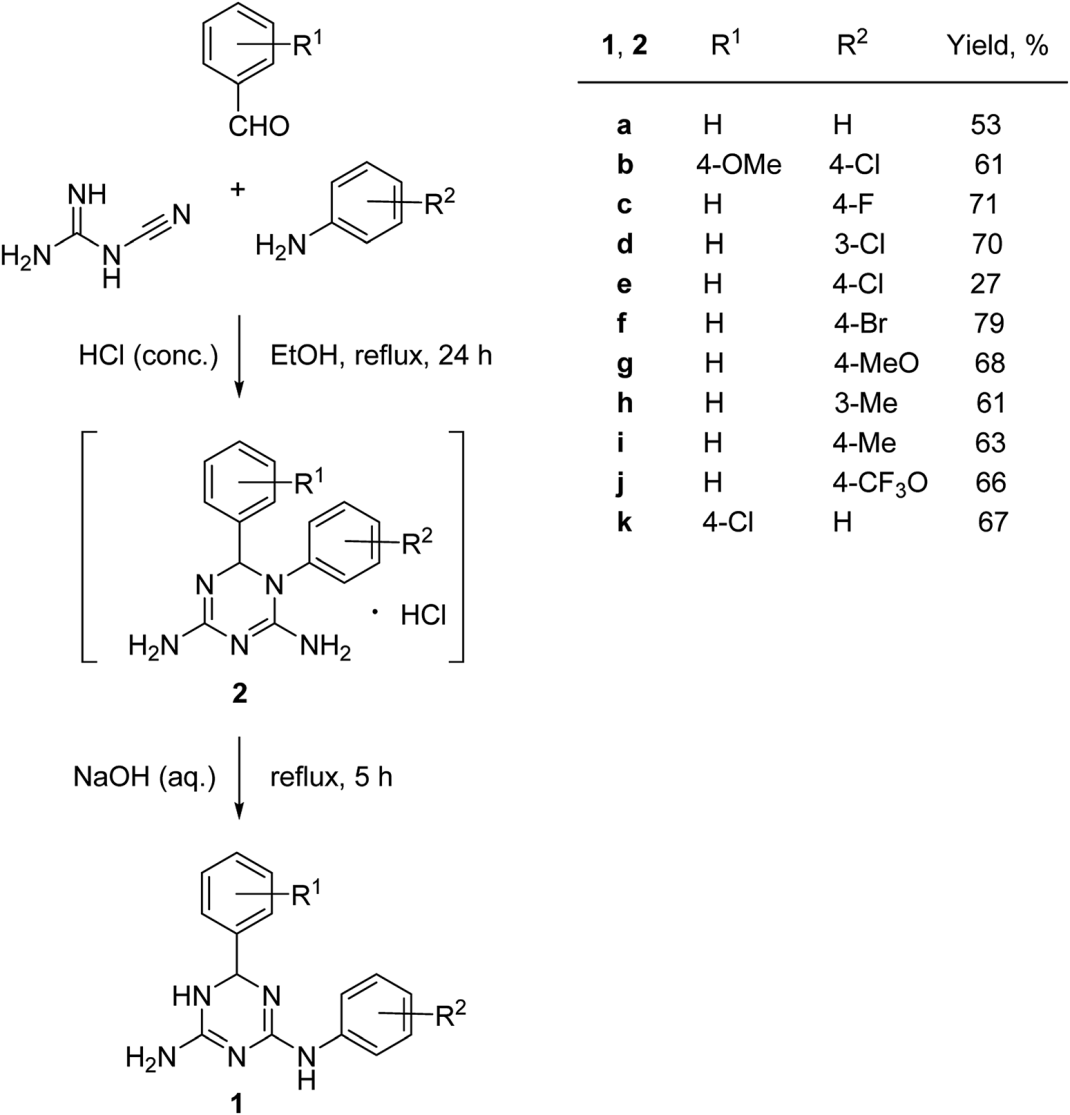
One-pot synthesis *N*^2^,6-diaryl-5,6-dihydro-1,3,5-triazine-2,4-diamines (1).

The synthesis of *N*^2^,6-diaryl-5,6-dihydro-1,3,5-triazine-2,4-diamines (2) was performed using a one-pot methodology combining two steps: (1) a three-component condensation of cyanoguanidine, an aniline and a benzaldehyde in the presence in hydrochloric acid and (2) a base-promoted Dimroth rearrangement of the intermediate 1,6-diaryl-1,6-dihydro-1,3,5-triazine-2,4-diamine hydrochlorides (2) (Scheme 2).

Variations of substituents on aniline (1c–j) benzaldehyde (1k), and their combinations (1b) were tolerated in the reaction. The structure of the prepared compounds 1 was confirmed by NMR spectroscopic data. The signals of the sp^3^-hydridized carbon atom in ^13^C NMR spectra at 67.5–68.4 ppm and the corresponding proton in ^1^H NMR spectra at 5.70–5.77 ppm confirmed formation of the dihydrotriazine ring. We found that it was critical for the success of the reaction to maintain pH at 10–11. Further increase of the pH resulted in the partial dehydrogenation of the product and aromatization of the dihydrotriazine in a process similar to the one we reported earlier.^5^

The products were isolated as a racemic mixture of *R*- and *S*-stereoisomers in the form of ethanol solvates after recrystallization from this solvent. The integration of signals at 1.06, 3.44–3.45, and 4.34–4.37 ppm, attributed to ethanol, in the ^1^H NMR spectra of samples dried in air at room temperature indicated that all prepared solvates of *N*^2^,6-diaryl-5,6-dihydro-1,3,5-triazine-2,4-diamines (1) contained one molecule of ethanol per heterocycle molecule. This was further confirmed by X-ray crystallographic studies as described below. We did not observe formation of stable clathrates with methanol and desolvated compounds 1 can be obtained by the recrystallization from this solvent. The NMR spectra of a representative desolvated sample of 1a can be found in ESI.†

**Scheme 3:**
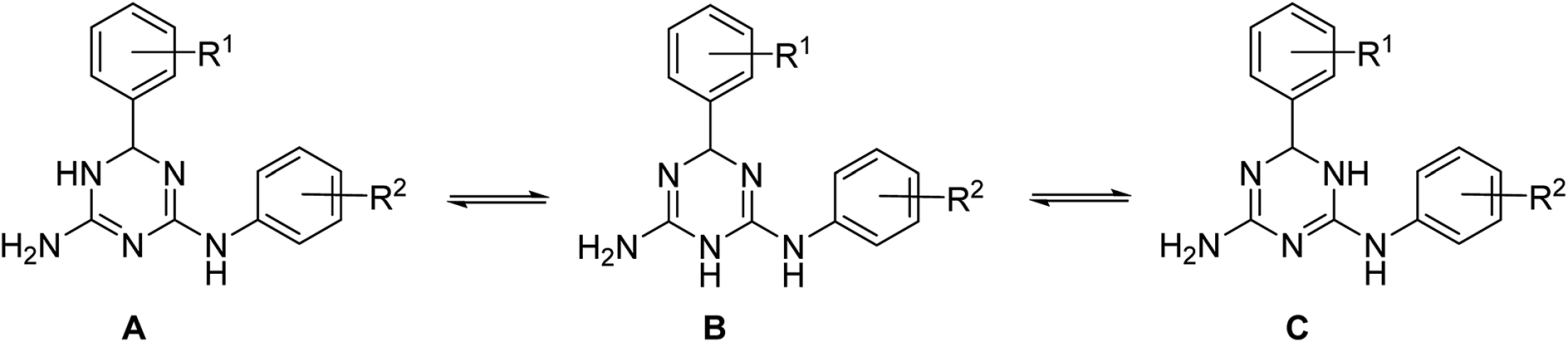
Annular tautomerism in *N*^2^,6-diaryl-X,6-dihydro-1,3,5-triazine-2,4-diamines.

In principle, three tautomeric forms of the prepared compounds are possible due to annular prototropic tautomerism in the dihydrotriazine ring (Scheme 3). In the ^1^H NMR spectra of compounds 1, signals of the annular NH and the NH link between the triazine and benzene rings were very broad and not always detectable. This can be attributed to the tautomerism, proton exchange with ethanol, or both processes taking place together. X-ray crystallography (*vide infra*) revealed that the compounds crystallized as the 5,6-dihydro-tautomer, A.

**Fig. 1:**
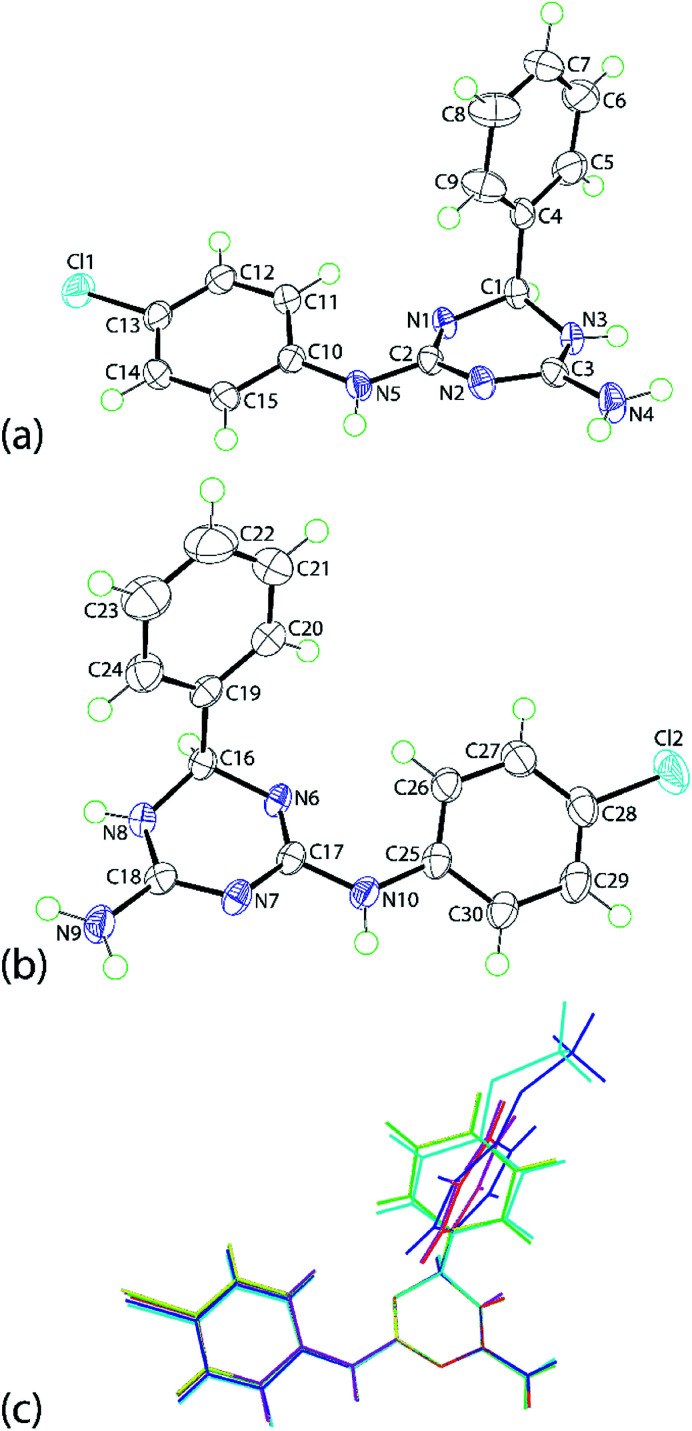
The molecular structures of the two independent organic molecules comprising the asymmetric unit of 1e, showing atom labelling scheme and displacement ellipsoids at the 70% probability level: (a) N1-containing molecule and (b) N6-containing molecule. (c) An overlay diagram of 1b (N1-molecule, blue image), 1b (inverted N6-molecule, aqua), 1e (N1-molecule, red), 1e (inverted N6-molecule, green), 1f (inverted N1-molecule, pink) and 1f (N6-molecule, yellow). The molecules have been overlapped so the N1, N2 and N3 atoms are coincident. Solvent ethanol molecules are omitted.

The molecular structures of 1b, 1e and 1f were established by single crystal X-ray crystallography as their ethanol solvates (1 : 1) and found to be isostructural. The discussion will focus on 1e·EtOH with details of 1b·EtOH and 1f·EtOH available in the ESI. Crystals of 1e·EtOH adopt the non-centrosymmetric space group *P*2_1_ with two independent molecules each of 1e and ethanol comprising the asymmetric unit of 1e·EtOH, Fig. 1; the molecular structure diagrams for 1b·EtOH and 1f·EtOH are given in ESI Fig. S1 and S2,† respectively.

The independent molecules of 1e are related by a non-crystallographic centre of inversion with the configurations at the C1 and C16 atoms being *R* and *S*, respectively; this therefore, an example of kyptoracemic behaviour.^7^ Key geometric parameters are collated in Table 1 for 1e·EtOH and ESI Table S1† for 1b·EtOH and 1f·EtOH. The parameters describing chemically equivalent bonds and angles follow the same trends across the series. At least three key parameters point to the adoption of 5,6-dihydro-tautomer, A, shown in Scheme 3. These are the short C2–N1 bond, consistent with significant double bond character, the wider angle subtended at the N3 atom compared with those at the N1 and N2 atoms, consistent with protonation at N3, and the participation of the N3-bound proton in significant hydrogen bonding interactions with the N7 atom of the second independent molecule and conversely, the N8-bound proton to the N2 atom. Based on the closeness of the C3–N2 and C3–N3 bond lengths, there is some evidence of delocalization of the π-electron density over these atoms.

**Table 1:** Key geometric parameters (Å, °) for 1e·EtOH

C1–N1	1.449(3)	C16–N6	1.458(3)
C1–N3	1.469(3)	C16–N8	1.456(3)
C2–N1	1.305(3)	C17–N6	1.300(3)
C2–N2	1.383(3)	C17–N7	1.378(3)
C3–N2	1.337(3)	C18–N7	1.330(3)
C3–N3	1.329(3)	C18–N8	1.339(3)
C1–N1–C2	115.67(19)	C16–N6–C18	114.63(19)
C2–N2–C3	114.0(2)	C17–N7–C18	114.4(2)
C1–N3–C3	118.5(2)	C16–N8–C18	118.1(2)
N1–C1–N3	110.70(19)	N6–C16–N8	110.92(19)
N1–C2–N2	127.6(2)	N6–C17–N7	127.5(2)
N2–C3–N3	122.9(2)	N7–C18–N8	122.6(2)

The N1-triazine ring adopts an envelope conformation whereby the C1 atom lies 0.442(3) Å out of the plane defined by the five remaining atoms, r.m.s. deviation = 0.033 Å; the equivalent parameters for the N6-ring/C16 atom are 0.471(3) and 0.034 Å, respectively. There are some conformational differences evident between the two independent molecules as highlighted in the overlay diagram of Fig. 1(c). These relate primarily to the relative orientation of the phenyl rings. The dihedral angles between the best plane through the triazine ring and the phenyl and chlorophenyl rings are 89.59(8) and 17.74(12)°, respectively for the N1-molecule; the equivalent angles for the N6-molecule are 72.76(9) and 19.24(14)°, respectively. For the N1- and N6-molecules, the dihedral angle between the peripheral rings are 77.95(8) and 57.86(9)°, respectively. Very similar trends are evident in the molecules comprising the methoxy (1b) and bromo (1f) analogues; see ESI Table S2† for data.

**Fig. 2:**
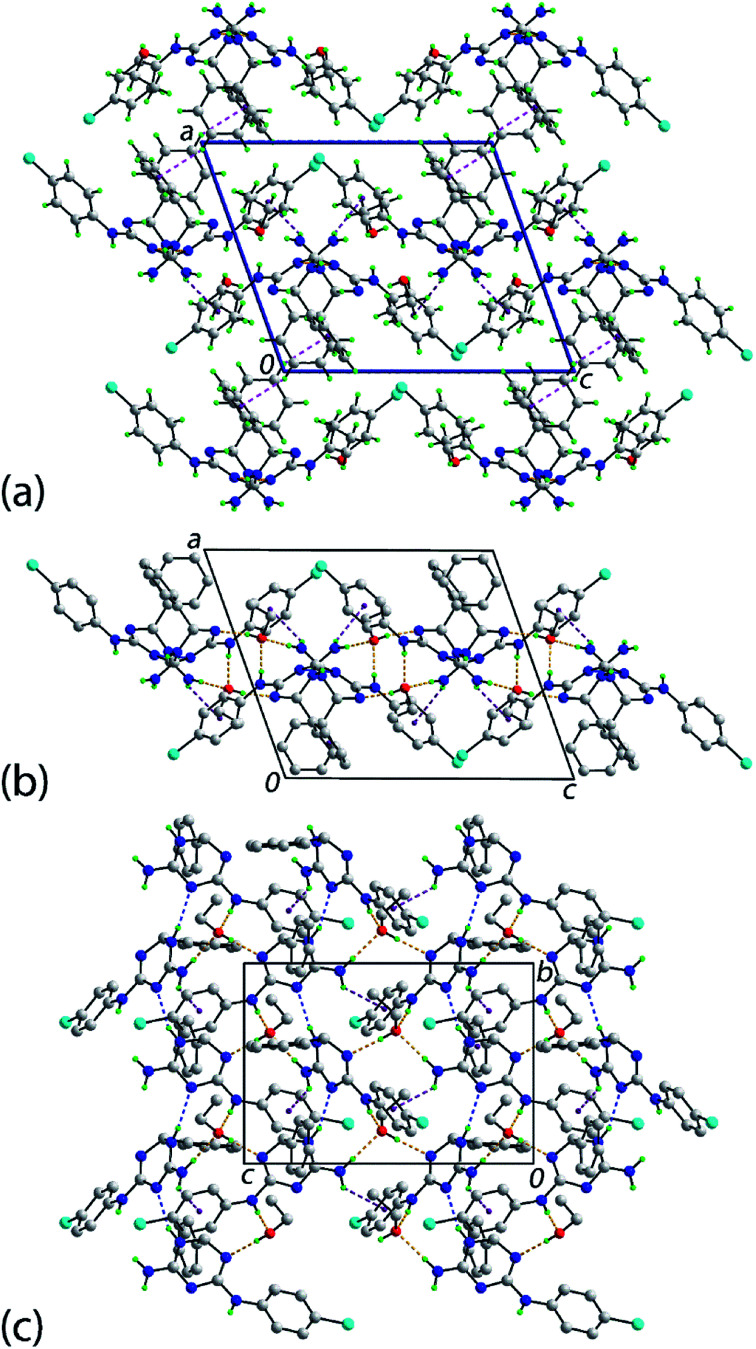
Molecular packing in the crystal of 1e·EtOH: (a) a view of the unit cell contents in projection down the *b*-axis. The N–H⋯N, N–H⋯π and C–H⋯π interactions are shown as blue, purple and pink dashed lines, respectively. (b) A side-on view of a supramolecular layer highlighting the encapsulation of the solvent ethanol molecules and (c) a plan view of the layer shown in (b). Hydrogen-bonding interactions involving ethanol are indicated by orange dashed lines. For (b) and (c), non-participating hydrogen atoms are omitted for reasons of clarity.

An interesting feature of the crystal structure determinations of 1b·EtOH, 1e·EtOH and 1f·EtOH is that each has occluded solvent in their structures so the ratio of organic molecule to ethanol was 1 : 1. A description of the molecular packing of 1e·EtOH ensues; details of the intermolecular interactions are gathered in Table 2. Fig. 2(a) shows a view of the molecular packing for 1e·EtOH showing only contacts occurring between the organic molecules, *i.e.*1e only. The two 1e molecules in the asymmetric unit are connected into helical supramolecular chains *via* ring amine-N–H⋯N(imine) hydrogen-bonding. The chains are aligned along the *b*-axis direction, being propagated by screw (2_1_) symmetry. The connections between chains along the *c*-axis are of the type primary amine-N–H⋯π(chlorophenyl), again occurring between the different 1e molecules of the asymmetric unit. Links between layers along the *a*-axis are of the type phenyl-C–H⋯π(phenyl). Here, the donor and acceptor molecules are the N1- and N6-containing molecules, respectively. As there is no reciprocal contact, *i.e.* having the N6- and N- molecules as the donor and acceptor, this interaction distinguishes the two independent molecules. Each of the independent ethanol molecules participates in analogous hydrogen-bonding with the 1e molecules defining the host structure. The donor interactions are of the type ethanol–O–H⋯N6(triazine), where the nitrogen acceptor is adjacent to the methine-carbon atom. The ethanol molecules each accept two hydrogen bonds, one from each of the exocyclic amines.

**Table 2:** Geometric parameters (Å, °) characterizing the intermolecular interactions occurring in the crystal of 1e·EtOH

A	H	B	A–H	H⋯B	A⋯B	A–H⋯B	Symmetry operation
N3	H3n	N7	0.87(2)	2.11(2)	2.931(3)	156(2)	1 − *x*, 1/2 + *y*, 1 − *z*
N8	H8n	N2	0.87(2)	2.12(2)	2.939(3)	156(2)	1 − *x*, 1/2 + *y*, 1 − *z*
N4	H4na	Cg(C10–C15)	0.88(2)	2.60(3)	135(2)	3.279(2)	*x*, *y*, *z*
N9	H9na	Cg(C25–C30)	0.87(2)	2.63(4)	128(3)	3.236(3)	*x*, *y*, 1 + *z*
C22	H22	Cg(C4–C9)	0.95	2.71	3.577(3)	152	1 + *x*, *y*, 1 + *z*
O1	H1o	N6	0.84(2)	1.88(2)	2.706(3)	169(3)	*x*, *y*, *z*
O2	H2o	N1	0.84(2)	1.89(2)	2.726(3)	172(3)	*x*, *y*, 1 + *z*
N4	H4nb	O1	0.88(3)	1.99(3)	2.865(3)	176(3)	*x*, *y*, *z*
N10	H10n	O1	0.88(2)	2.05(3)	2.913(3)	165(2)	1 − *x*, −1/2 + *y*, 1 − *z*
N5	H5n	O2	0.88(2)	2.04(2)	2.907(3)	169(2)	1 − *x*, −1/2 + *y*, 1 − *z*
N9	H9nb	O2	0.88(3)	2.00(3)	2.874(3)	177(3)	*x*, *y*, *z*

Subtle differences in the molecular packing are apparent in the crystal of 1b·EtOH; see ESI Table S3 and Fig. S3.† Crucially, the intra-layer O–H⋯N, N–H⋯O and N–H⋯N hydrogen-bonding remains the same. However, the connections between layers are chlorophenyl-C–H⋯O(methoxy) interactions. Additional intra-layer methoxyphenyl-C–H⋯π(chlorophenyl) and ethanol–methylene–C–H⋯Cl interactions are noted, which contribute to the stability of the layer. The molecular packing of the bromo analogue of 1e·EtOH, *i.e.*1f·EtOH, mimics that described above with the addition of inter-layer phenyl-C–H⋯Br contacts; see ESI Table S4 and Fig. S4.†

Additional investigations were conducted on the three crystallographically characterised compounds, *i.e*. simultaneous thermal analysis (STA) and powder X-ray diffraction (PXRD) studies. The original STA traces and data are given in ESI Fig. S5.† For 1b·EtOH, the results of the thermogravimetric analyses showed a single-step between 90 and 162 °C corresponding to the loss of one ethanol molecule per molecule of 1b. Similar features were noted for each of 1e·EtOH and 1f·EtOH although the onset temperatures were higher and the process was completed at lower temperatures. For 1e·EtOH and 1f·EtOH, a second exothermic event corresponding to melting was also observed. This was not seen in the STA of 1b·EtOH and visual inspection showed the sample to be a paste after desolvation in contrast to the powders generated after the desolvation of the other samples. In order to ascertain whether desolvation altered the crystal structures of 1e·EtOH and 1f·EtOH, PXRD measurements were conducted. As seen from ESI Fig. S6,† the desolvated forms did not retain the original crystal structures. Attempts at re-solvation of 1e and 1f were performed. Thus, powders were placed in a sample vial which was in turn placed in a larger container containing ethanol, capped and allow to stand overnight. Subsequent PXRD measurements showed no evidence of re-solvation.

## Conclusions

An efficient one-pot protocol for the synthesis of *N*^2^,6-diaryl-5,6-dihydro-1,3,5-triazine-2,4-diamines (1) from simple, readily available reagents *i.e.* cyanoguanidine, anilines and benzaldehydes was developed. After the initial three-component condensation of these building blocks, the intermediate 1,6-diaryl-1,6-dihydro-1,3,5-triazine-2,4-diamines (2) were isomerized to the targeted products *via* the base-promoted Dimroth rearrangement. The X-ray crystallographic study established the tautomeric form as the 5,6-dihydro-tautomer. It was found that *N*^2^,6-diaryl-5,6-dihydro-1,3,5-triazine-2,4-diamines (1) crystallised from ethanol trapping one molecule of this solvent per molecule of 1 in the crystals, giving 1·EtOH. The STA and PXRD data for selected compounds suggested that desolvation irreversibly change crystal structure.

## Experimental

### General information

All the chemicals and reagents were purchased from commercial suppliers (Sigma-Aldrich, Merck, and Alfa-Aesar) and were used without additional purification. Melting points (uncorrected) were determined on a Stuart™ SMP40 automatic melting point apparatus. ^1^H and ^13^C NMR spectra were recorded on a Bruker Fourier NMR spectrometer (300 MHz) using DMSO-*d*_6_ as a solvent and TMS as an internal reference. The chemical shifts are reported in parts per million (ppm, *δ*) and coupling constants are reported in Hertz with the splitting pattern described as singlet (s), broad signal (brs), doublet (d), triplet (t), quartet (q), doublet of doublets (dd), double of doublet of doublets (ddd), or multiplet (m). Thermogravimetric analyses were performed on a PerkinElmer STA 6000 Thermogravimetric Analyzer in the range 25–600 °C at the rate of 10 °C min^−1^ under a nitrogen purge. The data was manipulated by PerkinElmer thermal analysis proprietary software Pyris®. Powder X-ray diffraction (PXRD) patterns were measured on a Rigaku SmartLab X-ray diffractometer using Cu-Kα (*λ* = 1.5406 Å) radiation in the 2*θ* range 10 to 60° with step size 0.01°.

### General procedure for the preparation of *N*^2^,6-diaryl-5,6-dihydro-1,3,5-triazine-2,4-diamines (1)

To a solution of cyanoguanidine (0.84 g, 10 mmol), a (un)substituted benzaldehyde (10 mmol) and a (un)substituted aniline (10 mmol) in EtOH (5 mL), conc. HCl (0.84 mL, 10 mmol) was added and the reaction mixture was heated under reflux for 24 h. The reaction mixture was cooled to rt and diluted with 50% aq. EtOH (5 mL). Then, aq. NaOH solution (5 *N*) was added drop-wise to the solution until the pH increased to 10–11. The mixture was heated under reflux for another 5 h. After cooling at 0 °C, the resulting precipitate was filtered, washed with water and recrystallized from EtOH.

#### N^2^,6-Diphenyl-5,6-dihydro-1,3,5-triazine-2,4-diamine ethanolate (1a⋯EtOH)

Yield: 1.4 g, 53%; mp: 154–156 °C (EtOH). ^1^H NMR (300 MHz, DMSO-*d*_6_): *δ* 1.06 (3H, t, *J* = 7.0 Hz, CH_3_), 3.44 (2H, q, *J* = 7.0 Hz, CH_2_), 4.37 (1H, brs, OH), 5.75 (1H, s, CH), 5.84 (2H, brs, NH_2_), 6.73 (1H, t, *J* = 7.3 Hz, H-4′′), 7.10 (2H, t, *J* = 7.4 Hz, H-3′′ and H-5′′), 7.27–7.44 (5H, m, H-2′, H-3′, H-4′, H-5′ and H-6′), 7.73 (2H, d, *J* = 7.6 Hz, H-2′′ and H-6′′); ^13^C NMR (75 MHz, DMSO-*d*_6_): *δ* 18.5 (CH_3_), 55.9 (CH_2_), 68.2 (C-6), 117.9 (C-2′′ and C-6′′), 119.3 (C-4′′), 126.1 (C-3′ and C-5′), 127.2 (C-4′), 127.9 (C-3′′ and C-5′′), 128.1 (C-2′ and C-6′), 142.3 (C-1′), 145.7 (C-1′′), 155.4 (C-2), 157.9 (C-4). Anal. calcd for C_17_H_21_N_5_O: C, 65.57; H, 6.80; N, 22.49. Found: C, 65.44; H, 6.67; N, 22.55.

#### *N* ^2^-(4-Chlorophenyl)-6-(4-methoxyphenyl)-5,6-dihydro-1,3,5-triazine-2,4-diamine ethanolate (1b⋯EtOH)

Yield: 2.0 g, 61%; mp: 128–130 °C (EtOH). ^1^H NMR (300 MHz, DMSO-*d*_6_): *δ* 1.06 (3H, t, *J* = 7.0 Hz, CH_3_), 3.44 (2H, q, *J* = 7.0 Hz, CH_2_), 3.74 (3H, s, OCH_3_), 4.36 (1H, brs, OH), 5.70 (1H, s, CH), 5.81 (2H, brs, NH_2_), 6.91 (2H, d, *J* = 8.8 Hz, H-3′ and H-5′), 7.12 (2H, d, *J* = 9.0 Hz, H-3′′ and H-5′′), 7.32 (2H, d, *J* = 8.6 Hz, H-2′ and H-6′), 7.78 (2H, d, *J* = 8.9 Hz, H-2′′ and H-6′′); ^13^C NMR (75 MHz, DMSO-*d*_6_): *δ* 18.5 (CH_3_), 55.0 (OCH_3_), 55.9 (CH_2_), 67.7 (C-6), 113.4 (C-3′ and C-5′), 119.2 (C-2′′ and C-6′′), 122.5 (C-4′′), 127.3 (C-2′ and C-6′), 127.6 (C-3′′ and C-5′′), 137.8 (C-1′), 141.5 (C-1′′), 155.4 (C-2), 157.9 (C-4), 158.5 (C-4′). Anal. calcd for C_18_H_22_ClN_5_O_2_: C, 57.52; H, 5.90; N, 18.63. Found: C, 57.29; H, 5.76; N, 18.75.

#### *N* ^2^-(4-Fluorophenyl)-6-phenyl-5,6-dihydro-1,3,5-triazine-2,4-diamine ethanolate (1c⋯EtOH)

Yield: 2.0 g, 71%; mp: 202–204 °C (EtOH). ^1^H NMR (300 MHz, DMSO-*d*_6_): *δ* 1.06 (3H, t, *J* = 7.0 Hz, CH_3_), 3.45 (2H, q, *J* = 7.0 Hz, CH_2_), 4.35 (1H, brs, OH), 5.74 (1H, s, CH), 5.80 (2H, brs, NH_2_), 6.92 (2H, dd, ^3^*J*_HF_ = 9.0 Hz, ^3^*J*_HH_ = 9.0 Hz, H-3′′ and H-5′′), 7.25–7.30 (1H, m, H-4′), 7.33–7.43 (4H, m, H-2′, H-3′, H-5′ and H-6′), 7.76 (2H, dd, ^4^*J*_HF_ = 5.1 Hz, ^3^*J*_HH_ = 9.1 Hz, H-2′′ and H-6′′); ^13^C NMR (75 MHz, DMSO-*d*_6_): *δ* 18.5 (CH_3_), 56.0 (CH_2_), 68.3 (C-6), 114.2 (d, ^2^*J*_CF_ = 21.6 Hz, C-3′′ and C-5′′), 119.0 (d, ^3^*J*_CF_ = 7.1 Hz, C-2′′ and C-6′′), 126.2 (C-3′ and C-5′), 127.2 (C-4′), 128.1 (C-2′ and C-6′), 139.0 (d, ^4^*J*_CF_ = 1.5 Hz, C-1′′), 145.8 (C-1′), 155.8 (d, ^1^*J*_CF_ = 235.5 Hz, C-4′′), 155.4 (C-2), 157.9 (C-4). Anal. calcd for C_17_H_20_FN_5_O: C, 61.99; H, 6.12; N, 21.26. Found: C, 61.86; H, 5.98; N, 21.40.

#### *N* ^2^-(3-Chlorophenyl)-6-phenyl-5,6-dihydro-1,3,5-triazine-2,4-diamine ethanolate (1d⋯EtOH)

Yield: 2.1 g, 70%; mp: 154–156 °C (EtOH). ^1^H NMR (300 MHz, DMSO-*d*_6_): *δ* 1.06 (3H, t, *J* = 7.0 Hz, CH_3_), 3.45 (2H, q, *J* = 7.0 Hz, CH_2_), 4.34 (1H, brs, OH), 5.78 (1H, s, CH), 5.85 (2H, brs, NH_2_), 6.75 (1H, ddd, *J* = 0.8 Hz, *J* = 2.0 Hz, *J* = 7.9 Hz, H-4′′), 7.01 (1H, brs, NH), 7.10 (1H, dd, *J* = 8.1 Hz, *J* = 8.1 Hz, H-3′′), 7.26–7.44 (5H, m, H-2′, H-3′, H-4′, H-5′ and H-6′), 7.58 (1H, ddd, *J* = 0.9 Hz, *J* = 1.6 Hz, *J* = 8.6 Hz, H-2′′), 8.04 (1H, s, H-6′′), 8.11 (1H, brs, NH); ^13^C NMR (75 MHz, DMSO-*d*_6_): *δ* 18.5 (CH_3_), 56.0 (CH_2_), 68.2 (C-6), 116.2 (C-6′′), 117.0 (C-2′′), 118.6 (C-4′′), 126.1 (C-3′ and C-5′), 127.3 (C-4′), 128.1 (C-2′ and C-6′), 129.3 (C-3′′), 132.4 (C-5′′), 144.1 (C-1′′), 145.6 (C-1′), 155.5 (C-2), 157.9 (C-4). Anal. calcd for C_17_H_20_ClN_5_O: C, 59.04; H, 5.83; N, 20.25. Found: C, 58.89; H, 5.67; N, 20.42.

#### *N* ^2^-(4-Chlorophenyl)-6-phenyl-5,6-dihydro-1,3,5-triazine-2,4-diamine ethanolate (1e⋯EtOH)

Yield: 0.8 g, 27%; mp: 213–215 °C (EtOH). ^1^H NMR (300 MHz, DMSO-*d*_6_): *δ* 1.06 (3H, t, *J* = 7.0 Hz, CH_3_), 3.44 (2H, q, *J* = 7.0 Hz, CH_2_), 4.34 (1H, brs, OH), 5.76 (1H, s, CH), 5.81 (2H, brs, NH_2_), 6.98 (1H, brs, NH), 7.12 (2H, d, *J* = 9.0 Hz, H-3′′ and H-5′′), 7.24–7.43 (5H, m, H-2′, H-3′, H-4′, H-5′ and H-6′), 7.78 (2H, d, *J* = 8.9 Hz, H-2′′ and H-6′′), 8.04 (1H, brs, NH); ^13^C NMR (75 MHz, DMSO-*d*_6_): *δ* 18.5 (CH_3_), 55.9 (CH_2_), 68.3 (C-6), 119.2 (C-2′′ and C-6′′), 122.5 (C-4′′), 126.1 (C-3′ and C-5′), 127.2 (C-4′), 127.6 (C-3′′ and C-5′′), 128.1 (C-2′ and C-6′), 141.5 (C-1′′), 145.6 (C-1′), 155.4 (C-2), 157.9 (C-4). Anal. calcd for C_17_H_20_ClN_5_O: C, 59.04; H, 5.83; N, 20.25. Found: C, 58.92; H, 5.72; N, 20.32.

#### *N* ^2^-(4-Bromophenyl)-6-phenyl-5,6-dihydro-1,3,5-triazine-2,4-diamine ethanolate (1f⋯EtOH)

Yield: 2.7 g, 79%; mp: 227–228 °C (EtOH). ^1^H NMR (300 MHz, DMSO-*d*_6_): *δ* 1.06 (3H, t, *J* = 7.0 Hz, CH_3_), 3.44 (2H, q, *J* = 7.0 Hz, CH_2_), 4.34 (1H, brs, OH), 5.75 (1H, s, CH), 5.82 (2H, brs, NH_2_), 6.98 (1H, brs, NH), 7.24 (2H, d, *J* = 9.0 Hz, H-3′′ and H-5′′), 7.27–7.43 (5H, m, H-2′, H-3′, H-4′, H-5′ and H-6′), 7.74 (2H, d, *J* = 8.9 Hz, H-2′′ and H-6′′), 8.03 (H, brs, NH); ^13^C NMR (75 MHz, DMSO-*d*_6_): *δ* 18.5 (CH_3_), 56.0 (CH_2_), 68.3 (C-6), 110.4 (C-4′′), 119.7 (C-2′′ and C-6′′), 126.1 (C-3′ and C-5′), 127.3 (C-4′), 128.1 (C-2′ and C-6′), 130.5 (C-3′′ and C-5′′), 141.9 (C-1′′), 145.6 (C-1′), 155.4 (C-2), 157.9 (C-4). Anal. calcd for C_17_H_20_BrN_5_O: C, 52.32; H, 5.17; N, 17.94. Found: C, 52.20; H, 5.05; N, 18.06.

#### *N* ^2^-(4-Methoxyphenyl)-6-phenyl-5,6-dihydro-1,3,5-triazine-2,4-diamine ethanolate (1g⋯EtOH)

Yield: 2.0 g, 68%; mp: 135–136 °C (EtOH). ^1^H NMR (300 MHz, DMSO-*d*_6_): *δ* 1.06 (3H, t, *J* = 7.0 Hz, CH_3_), 3.44 (2H, q, *J* = 7.0 Hz, CH_2_), 3.65 (3H, s, OCH_3_), 4.34 (1H, brs, OH), 5.72 (1H, s, CH), 5.74 (2H, brs, NH_2_), 6.70 (2H, d, *J* = 9.1 Hz, H-3′′ and H-5′′), 7.24–7.43 (5H, m, H-2′, H-3′, H-4′, H-5′ and H-6′), 7.64 (2H, d, *J* = 9.0 Hz, H-2′′ and H-6′′); ^13^C NMR (75 MHz, DMSO-*d*_6_): *δ* 18.5 (CH_3_), 55.0 (OCH_3_), 55.9 (CH_2_), 68.4 (C-6), 113.2 (C-3′′ and C-5′′), 119.1 (C-2′′ and C-6′′), 126.2 (C-3′ and C-5′), 127.1 (C-4′), 128.0 (C-2′ and C-6′), 135.8 (C-1′′), 145.9 (C-1′), 152.6 (C-4′′), 155.3 (C-2), 157.8 (C-4). Anal. calcd for C_18_H_23_N_5_O_2_: C, 63.32; H, 6.79; N, 20.51. Found: C, 63.11; H, 6.63; N, 20.72.

#### *N* ^2^-(3-Methylphenyl)-6-phenyl-5,6-dihydro-1,3,5-triazine-2,4-diamine ethanolate (1h⋯EtOH)

Yield: 1.7 g, 61%; mp: 155–157 °C (EtOH). ^1^H NMR (300 MHz, DMSO-*d*_6_): *δ* 1.06 (3H, t, *J* = 7.0 Hz, CH_3_), 2.18 (3H, s, CH_3_), 3.45 (2H, q, *J* = 7.0 Hz, CH_2_), 4.37 (1H, brs, OH), 5.76 (1H, s, CH), 5.85 (2H, brs, NH_2_), 6.56 (1H, d, *J* = 9.0 Hz, H-4′′), 6.98 (1H, dd, *J* = 7.8 Hz, *J* = 7.8 Hz, H-3′′), 7.27 (1H, t, *J* = 7.1 Hz, H-4′), 7.36 (2H, dd, *J* = 7.3 Hz, *J* = 7.3 Hz, H-3′ and H-5′), 7.42 (2H, dd, *J* = 1.2 Hz, *J* = 8.1 Hz, H-2′ and H-6′), 7.48 (1H, s, H-6′′), 7.60 (1H, d, *J* = 8.1 Hz, H-2′′), 6.5–8.03 (2H, brs, 2(NH)); ^13^C NMR (75 MHz, DMSO-*d*_6_): *δ* 18.5 (CH_3_), 21.3 (CH_3_), 55.9 (CH_2_), 68.2 (C-6), 115.2 (C-6′′), 118.4 (C-2′′), 120.1 (C-4′′), 126.1 (C-3′ and C-5′), 127.2 (C-4′), 127.7 (C-3′′), 128.0 (C-2′ and C-6′), 136.7 (C-5′′), 142.2 (C-1′′), 145.7 (C-1′), 155.4 (C-2), 157.8 (C-4). Anal. calcd for C_18_H_23_N_5_O: C, 66.44; H, 7.12; N, 21.52. Found: C, 66.29; H, 6.98; N, 21.69.

#### *N* ^2^-(4-Methylphenyl)-6-phenyl-5,6-dihydro-1,3,5-triazine-2,4-diamine ethanolate (1i⋯EtOH)

Yield: 1.8 g, 63%; mp: 206–208 °C (EtOH). ^1^H NMR (300 MHz, DMSO-*d*_6_): *δ* 1.06 (3H, t, *J* = 7.0 Hz, CH_3_), 2.17 (3H, s, CH_3_), 3.44 (2H, q, *J* = 7.0 Hz, CH_2_), 4.37 (1H, brs, OH), 5.74 (1H, s, CH), 5.91 (2H, brs, NH_2_), 6.91 (2H, d, *J* = 8.3 Hz, H-3′′ and H-5′′), 7.24–7.43 (4H, m, H-2′, H-3′, H-4′, H-5′ and H-6′), 7.60 (2H, d, *J* = 8.4 Hz, H-2′′ and H-6′′); ^13^C NMR (75 MHz, DMSO-*d*_6_): *δ* 18.5 (CH_3_), 20.2 (CH_3_), 55.9 (CH_2_), 68.3 (C-6), 117.9 (C-2′′ and C-6′′), 126.1 (C-3′ and C-5′), 127.2 (C-4′), 127.8 (C-4′′), 127.9 (C-2′ and C-6′), 128.0 (C-3′′ and C-5′′), 139.8 (C-1′), 145.8 (C-1′′), 155.4 (C-2), 157.8 (C-4). Anal. calcd for C_18_H_23_N_5_O: C, 66.44; H, 7.12; N, 21.52. Found: C, 66.28; H, 6.95; N, 21.74.

#### 6-Phenyl-*N*^2^-(4-(trifluoromethoxy)phenyl)-5,6-dihydro-1,3,5-triazine-2,4-diamine ethanolate (1j⋯EtOH)

Yield: 2.3 g, 66%; mp: 205–208 °C (EtOH). ^1^H NMR (300 MHz, DMSO-*d*_6_): *δ* 1.06 (3H, t, *J* = 7.0 Hz, CH_3_), 3.45 (2H, q, *J* = 7.0 Hz, CH_2_), 4.36 (1H, brs, OH), 5.77 (1H, s, CH), 5.85 (2H, brs, NH_2_), 7.09 (2H, d, *J* = 8.4 Hz, H-3′′ and H-5′′), 7.25–7.44 (5H, m, H-2′, H-3′, H-4′, H-5′ and H-6′), 7.85 (2H, d, *J* = 9.1 Hz, H-2′′ and H-6′′), 7.00–8.03 (2H, brs, 2(NH)); ^13^C NMR (75 MHz, DMSO-*d*_6_): *δ* 18.5 (CH_3_), 56.0 (CH_2_), 68.3 (C-6), 118.6 (C-3′′ and C-5′′), 120.2 (q, *J* = 254.6 Hz, OCF_3_), 120.8 (C-2′′ and C-6′′), 126.2 (C-3′ and C-5′), 127.3 (C-4′), 128.1 (C-2′ and C-6′), 140.7 (q, *J* = 1.5 Hz, C-4′′), 141.9 (C-1′′), 145.7 (C-1′), 155.5 (C-2), 158.0 (C-4). Anal. calcd for C_18_H_20_F_3_N_5_O_2_: C, 54.68; H, 5.10; N, 17.71. Found: C, 54.51; H, 4.97; N, 17.88.

#### 6-(4-Chlorophenyl)-*N*^2^-phenyl-5,6-dihydro-1,3,5-triazine-2,4-diamine ethanolate (1k⋯EtOH)

Yield: 2.0 g, 67%; mp: 182–185 °C (EtOH). ^1^H NMR (300 MHz, DMSO-*d*_6_): *δ* 1.06 (3H, t, *J* = 7.0 Hz, CH_3_), 3.44 (2H, q, *J* = 7.0 Hz, CH_2_), 4.35 (1H, brs, OH), 5.76 (1H, s, CH), 5.91 (2H, brs, NH_2_), 6.74 (1H, t, *J* = 7.3 Hz, H-4′′), 7.10 (2H, t, *J* = 7.9 Hz, H-3′′ and H-5′′), 7.43 (4H, m, H-2′, H-3′, H-5′ and H-6′), 7.72 (2H, d, *J* = 7.6 Hz, H-2′′ and H-6′′), 6.50–8.00 (2H, brs, 2(NH)); ^13^C NMR (75 MHz, DMSO-*d*_6_): *δ* 18.5 (CH_3_), 55.9 (CH_2_), 67.5 (C-6), 117.9 (C-2′′ and C-6′′), 119.4 (C-4′′), 127.9 (C-3′′ and C-5′′), 128.1 (C-2′ and C-6′), 128.0 (C-3′ and C-5′), 131.7 (C-4′), 142.2 (C-1′), 144.6 (C-1′′), 155.4 (C-2), 157.7 (C-4). Anal. calcd for C_17_H_20_ClN_5_O: C, 59.04; H, 5.83; N, 20.25. Found: C, 58.92; H, 5.69; N, 20.38.

### X-ray crystallographic analysis

X-ray intensity data for 1b·EtOH, 1e·EtOH and 1f·EtOH were measured at *T* = 100 K on Rigaku/Oxford Diffraction XtaLAB Synergy diffractometer (Dualflex, AtlasS2) fitted with Cu-Kα radiation (*λ* = 1.54178 Å) so that *θ*_max_ = 67.1°. Data reduction, including absorption correction, was accomplished with CrysAlisPro.^8^ The structures were solved by direct-methods^9^ and refined (anisotropic displacement parameters and C-bound H atoms in the riding model approximation) on *F*^2^.^10^ The O- and N-bound H atoms were refined with O–H and N–H constrained to 0.84 ± 0.01 and 0.88 ± 0.01 Å, respectively, and with *U*_iso_(H) = 1.2*U*_eq_(O, N). A weighting scheme of the form *w* = 1/[*σ*^2^(*F*_o_^2^) + (*aP*)^2^ + *bP*] where *P* = (*F*_o_^2^ + 2*F*_c_^2^)/3 was introduced in each case. The absolute structures were determined based on differences in Friedel pairs included in the respective data sets. In 1b·EtOH, two reflections, *i.e.* (3–5 8) and (3 5 8), were omitted from the final cycles of refinement owing to poor agreement. The molecular structure diagrams were generated with ORTEP for Windows^11^ with 50% displacement ellipsoids, and the packing diagrams were drawn with DIAMOND.^12^ Additional data analysis was made with PLATON.^13^ Crystal data and refinement details are given in Table 3.

**Table 3:** Crystallographic data and refinement details for 1b·EtOH, 1e·EtOH, and 1f·EtOH

Compound	1b·EtOH	1e·EtOH	1f·EtOH
Formula	C_16_H_16_ClN_5_O·C_2_H_6_O	C_15_H_14_ClN_5_·C_2_H_6_O	C_15_H_14_BrN_5_·C_2_H_6_O
Molecular weight	375.85	345.83	390.29
Crystal size/mm^3^	0.04 × 0.07 × 0.13	0.03 × 0.06 × 0.06	0.02 × 0.08 × 0.11
Colour	Colourless	Colourless	Colourless
Crystal system	Monoclinic	Monoclinic	Monoclinic
Space group	*P*2_1_	*P*2_1_	*P*2_1_
*a*/Å	13.2045(2)	12.6684(2)	12.7568(4)
*b*/Å	9.7903(2)	9.81230(10)	9.7768(3)
*c*/Å	14.8676(2)	15.0780(2)	15.1645(4)
*β*/°	93.816(2)	109.8020(10)	109.622(3)
*V*/Å^3^	1917.76(6)	1763.46(4)	1781.49(10)
*Z*	4	4	4
*D*_c_/g cm^−3^	1.302	1.303	1.455
*μ*/mm^−1^	1.949	2.030	3.258
Measured data	24 367	42 215	22 035
*θ* range/°	3.0–67.1	3.1–67.1	3.1–67.1
Unique data	6693	6286	5907
Observed data (*I* ≥ 2.0*σ*(*I*))	6456	6062	5554
No. parameters	503	465	465
*R*, obs. data; all data	0.053; 0.145	0.029; 0.072	0.037; 0.093
*a*; *b* in weighting scheme	0.071; 3.036	0.041; 0.308	0.058; 0.019
*R*_w_, obs. data; all data	0.055; 0.146	0.031; 0.073	0.040; 0.096
Range of residual electron			
Density peaks/eÅ^−3^	−0.46 to 0.60	−0.27 to 0.18	−0.74 to 0.74

## Conflicts of interest

There are no conflicts to declare.

## Supplementary Material

RA-009-C9RA08795H-s001

RA-009-C9RA08795H-s002
