# Supplementary material for: A one-pot synthesis of N2,6-diaryl-5,6-dihydro-1,3,5-triazine-2,4-diamines and systematic evaluation of their ability to host ethanol in crystals
Source: RSC Adv. 2019 Nov 19;9(64):37660–7. doi: 10.1039/c9ra08795h (PMC9075762; doi:10.1039/c9ra08795h)

Electronic Supplementary Material (ESI) for RSC Advances.
This journal is © The Royal Society of Chemistry 2019

RSC Advances

ELECTRONIC SUPPLEMENTARY INFORMATION

A one-pot synthesis of N^2,6-diaryl-5,6-dihydro-1,3,5-triazine-2,4-diamines and systematic evaluation of their ability to host ethanol in crystals

Ahmad Junaid,[a] Yee Seng Tan,[b] Edward R. T. Tiekink,[b] and Anton V. Dolzhenko[a,c]

[a] School of Pharmacy, Monash University Malaysia, Jalan Lagoon Selatan, Bandar Sunway, Selangor Darul Ehsan 47500, Malaysia

[b] Research Centre for Crystalline Materials, School of Science and Technology, 5 Jalan Universiti, Sunway University, Bandar Sunway, Selangor Darul Ehsan 47500, Malaysia

[c] School of Pharmacy and Biomedical Sciences, Curtin Health Innovation Research Institute, Faculty of Health Sciences, Curtin University, GPO Box U1987 Perth, Western Australia 6845, Australia

Table S1. Key geometric parameters (Å, °) for **1b**.EtOH and **1f**.EtOH

Parameter		Parameter		Parameter		Parameter	
**1b – molecule 1**		**1b – molecule 2**		**1f – molecule 1**		**1f – molecule 2**	
C1–N1	1.447(6)	C17–N6	1.471(6)	C1–N1	1.448(6)	C16–N6	1.463(6)
C1–N3	1.470(6)	C17–N8	1.452(6)	C1–N3	1.474(6)	C16–N8	1.452(6)
C2–N1	1.305(6)	C18–N6	1.312(7	C2–N1	1.301(7)	C17–N6	1.297(7)
C2–N2	1.373(6)	C18–N7	1.366(6)	C2–N2	1.379(6)	C17–N7	1.388(6)
C3–N2	1.339(6)	C19–N7	1.340(6)	C3–N2	1.338(6)	C18–N7	1.318(6)
C3–N3	1.334(6)	C19–N8	1.342(7)	C3–N3	1.324(7)	C18–N8	1.336(7)
C1–N1–C2	115.1(4)	C17–N6–C18	113.3(4)	C1–N1–C2	115.6(4)	C16–N6–C18	114.6(4)
C2–N2–C3	114.0(4)	C18–N7–C19	115.1(4)	C2–N2–C3	113.6(4)	C17–N7–C18	114.9(4)
C1–N3–C3	117.6(4)	C17–N8–C19	117.8(4)	C1–N3–C3	118.4(4)	C16–N8–C18	118.2(4)
N1–C1–N3	110.5(4)	N6–C17–N8	110.0(4)	N1–C1–N3	110.5(4)	N6–C16–N8	111.0(4)
N1–C2–N2	127.8(4)	N6–C18–N7	127.4(4)	N1–C2–N2	128.1(4)	N6–C17–N7	126.9(4)
N2–C3–N3	122.5(4)	N7–C19–N8	121.0(4)	N2–C3–N3	123.3(4)	N7–C18–N8	122.6(4)

Table S2. Least-squares plane data for **1b**.EtOH and **1f**.EtOH.

**1b**	
N1-molecule	
r.m.s. deviation (N1-N3, C2 and C3)	0.042
deviation of C1	0.477(6)
Dihedral angles	
(N1-N3, C1-C3)/(C4-C9)	89.07(13)
(N1-N3, C1-C3)/(C11-C16)	19.2(3)
(C4-C9)/(C11-C16)	70.43(15)
N6-molecule	
r.m.s. deviation (N6-N8, C17 and C8)	0.043
deviation of C16	0.529(6)
Dihedral angles	
(N6-N8, C16-C18)/(C20-C25)	74.56(17)
(N6-N8, C16-C18)/(C27-C32)	13.6(3)
(C20-C25)/(C27-C32)	63.38(17)
**1f**	
N1-molecule	
r.m.s. deviation (N1-N3, C2 and C3)	0.030
deviation of C1	0.441(6)
Dihedral angles	
(N1-N3, C1-C3)/(C4-C9)	88.31(16)
(N1-N3, C1-C3)/(C10-C15)	18.3(2)
(C4-C9)/(C10-C15)	75.81(17)
N6-molecule	
r.m.s. deviation (N6-N8, C17 and C8)	0.032
deviation of C16	0.467(6)
Dihedral angles	
(N6-N8, C16-C18)/(C19-C24)	72.61(18)
(N6-N8, C16-C18)/(C25-C30)	19.3(3)
(C19-C24)/(C25-C30)	57.61(18)

Table S3 Geometric parameters (Å, °) characterizing the intermolecular interactions occurring in the crystal of **1b**.EtOH

A	H	B	A–H	H···B	A···B	A–H···B	Symmetry operation
N3	H3n	N7	0.88(4)	2.08(4)	2.932(6)	161(4)	1-*x*, ½+*y*, 1-*z*
N8	H8n	N2	0.88(4)	2.08(3)	2.929(6)	161(5)	1-*x*, ½+*y*, 1-*z*
N4	H4na	Cg(C27-C32)	0.88(4)	2.59(5)	137(4)	3.290(5)	*x*, *y*, 1+*z*
N9	H9na	Cg(C11-C16)	0.88(5)	2.72(5)	134(4)	3.389(5)	*x*, *y*, *z*
C22	H22	Cg(C11-C16)	0.95	2.76	3.565(6)	143	1-*x*, 1/2+*y*, 1-*z*
O3	H3o	N1	0.88(5)	1.86(5)	2.712(5)	162(5)	*x*, *y*, *z*
O4	H4o	N6	0.87(5)	1.88(5)	2.732(5)	167(5)	*x*, *y*, 1+*z*
N5	H5n	O3	0.88(4)	2.05(4)	2.916(6)	167(5)	1-*x*, -½+*y*, 1-*z*
N9	H9Nb	O3	0.88(5)	2.03(5)	2.861(6)	158(6)	*x*, *y*, *z*
N4	H4nb	O4	0.88(5)	2.02(5)	2.883(6)	170(5)	*x*, *y*, *z*
N10	H10n	O4	0.88(4)	2.07(4)	2.912(6)	163(5)	1-*x*, -½+*y*, 1-*z*
C15	H15	O2	0.95	2.44	3.275(7)	147	-1+ *x*, *y*, *z*
C29	H29	O1	0.95	2.42	3.342(7)	165	1-*x*, ½+*y*, -*z*
C33	H33a	Cl2	0.99	2.80	3.551(7)	133	1-*x*, ½+*y*, -*z*

Table S4 Geometric parameters (Å, °) characterizing the intermolecular interactions occurring in the crystal of **1f**.EtOH

A	H	B	A–H	H···B	A···B	A–H···B	Symmetry operation
N3	H3n	N7	0.88(3)	2.04(3)	2.911(6)	168(4)	1-*x*, -½+*y*, 1-*z*
N8	H8n	N2	0.88(4)	2.10(4)	2.943(6)	161(5)	1-*x*, -½+*y*, 1-*z*
N4	H4na	Cg(C25-C30)	0.89(4)	2.58(6)	136(4)	3.279(5)	*x*, *y*, 1+*z*
N9	H9na	Cg(C10-C15)	0.88(3)	2.65(6)	127(5)	3.257(6)	*x*, *y*, *z*
C22	H22	Cg(C4-C9)	0.95	2.75	3.611(6)	152	*x*, *y*, 1+*z*
O1	H1o	N1	0.84(5)	1.89(5)	2.728(5)	175(5)	*x*, *y*, *z*
O2	H2o	N6	0.83(5)	1.88(5)	2.708(5)	173(7)	*x*, *y*, *z*
N5	H5n	O1	0.88(5)	2.02(5)	2.890(5)	172(5)	1-*x*, ½+*y*, 1-*z*
N9	H9nb	O1	0.88(5)	2.00(5)	2.878(6)	177(6)	*x*, *y*, *z*
N4	H4nb	O2	0.87(5)	2.02(5)	2.885(6)	173(4)	*x*, *y*, 1+*z*
N10	H10n	O2	0.88(5)	2.03(5)	2.894(6)	170(5)	1-*x*, ½+*y*, *z*
C7	H7	Br1	0.95	2.92	3.682(7)	138	-*x*, -½+*y*, 1-*z*

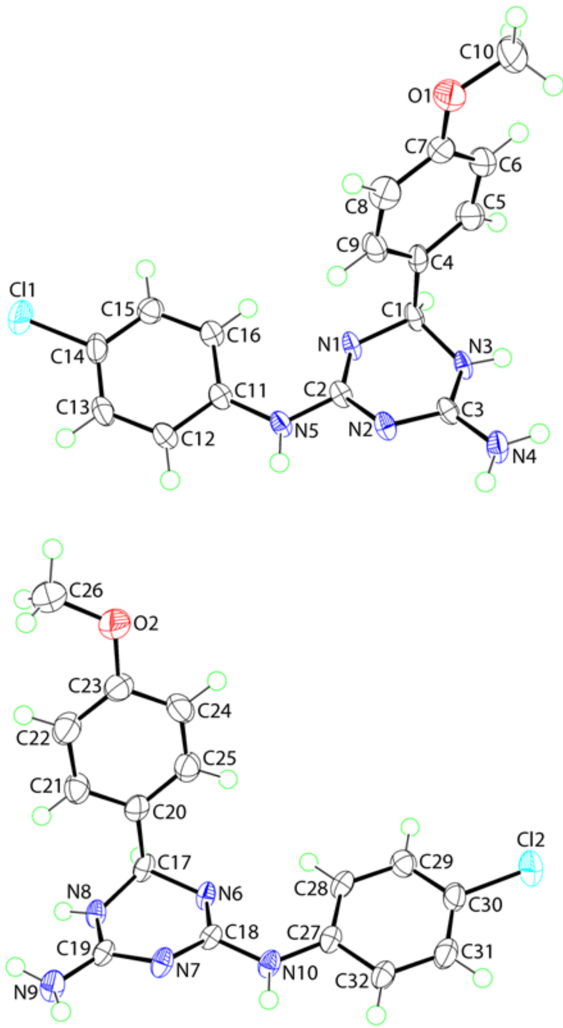

Figure S1. The molecular structures of the two independent molecules of **1b** comprising the asymmetric unit of **1b**.EtOH, showing atom labelling scheme and displacement ellipsoids at the 70% probability level.

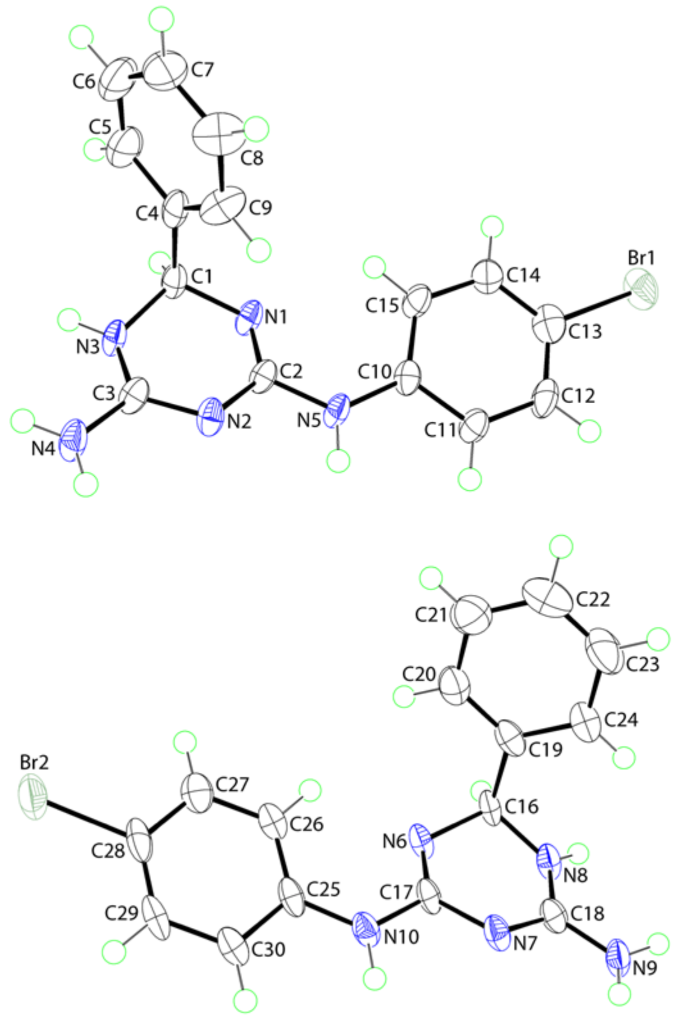

Figure S2. The molecular structures of the two independent molecules of **1e** comprising the asymmetric unit of **1e**.EtOH, showing atom labelling scheme and displacement ellipsoids at the 70% probability level.

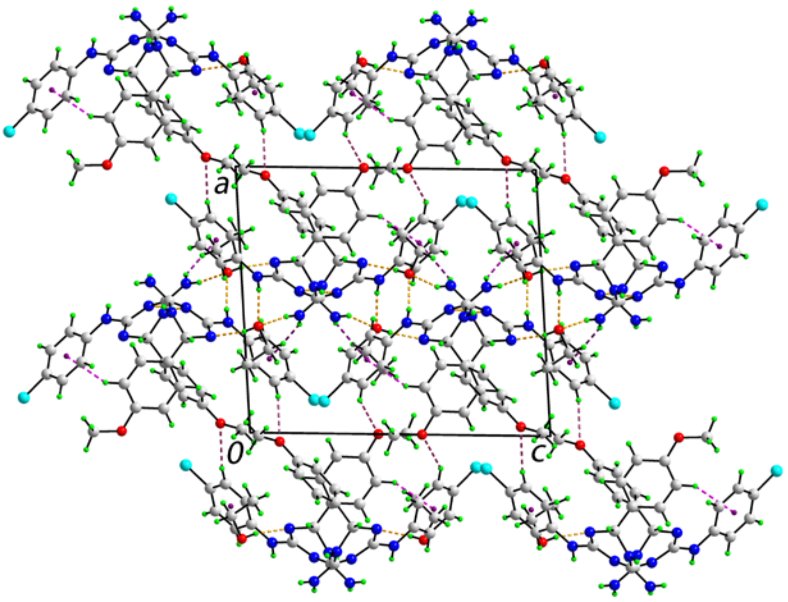

(a)

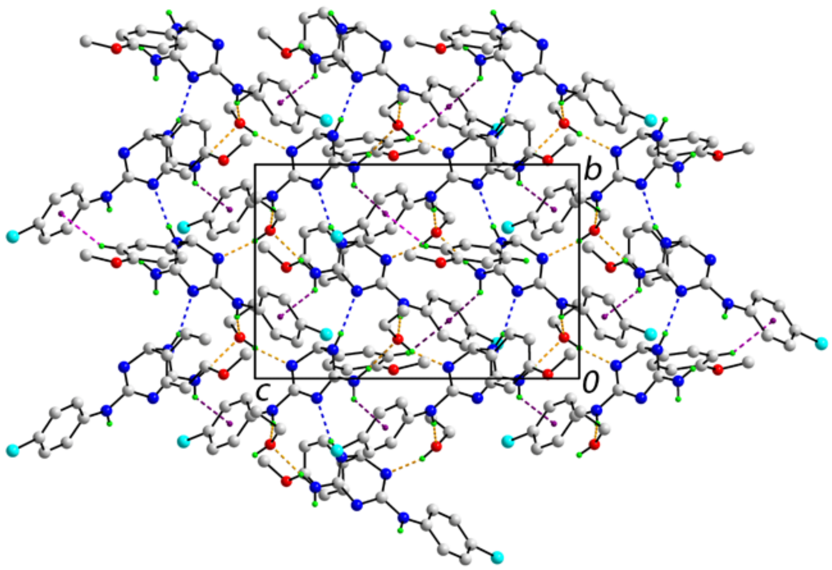

(b)

Figure S3. Molecular packing in **1b**.EtOH: (a) view of the unit cell contents in projection down the *a*-axis highlighting the stacking of layers and (b) a plan view of the layer (non-participating hydrogen atoms are omitted for reasons of clarity). The N–H···N, N–H···π, C–H···π an d C–H···O interactions are shown as blue, purple, pink and plum dashed lines, respectively. The hydrogen-bonding involving the ethanol molecules are shown as orange dashed lines.

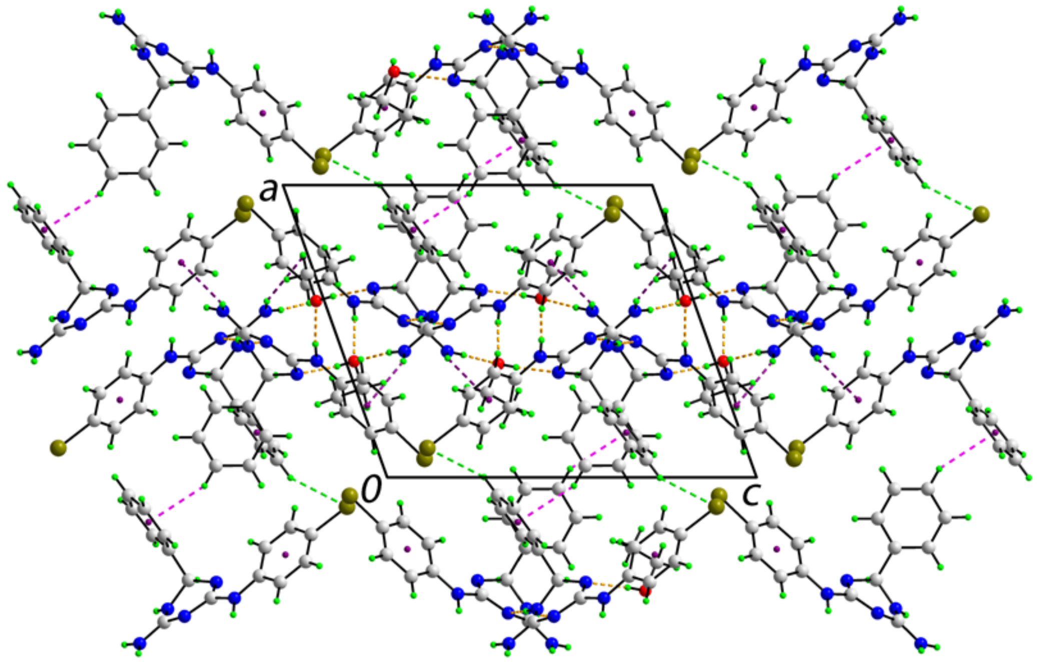

Figure S4. Molecular packing in **1f**.EtOH: view of the unit cell contents in projection down the *b*-axis highlighting the stacking of layers. The N–H···N, N–H···π, C–H···π and C–H···Br interactions are shown as blue, purple, pink and green dashed lines, respectively. Hydrogen-bonding involving the ethanol molecules are shown as orange dashed lines.

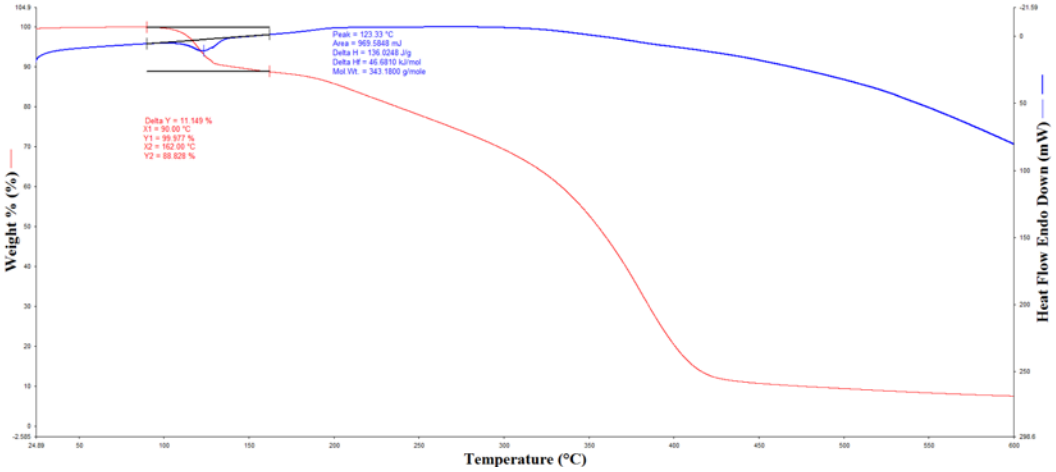

(a)

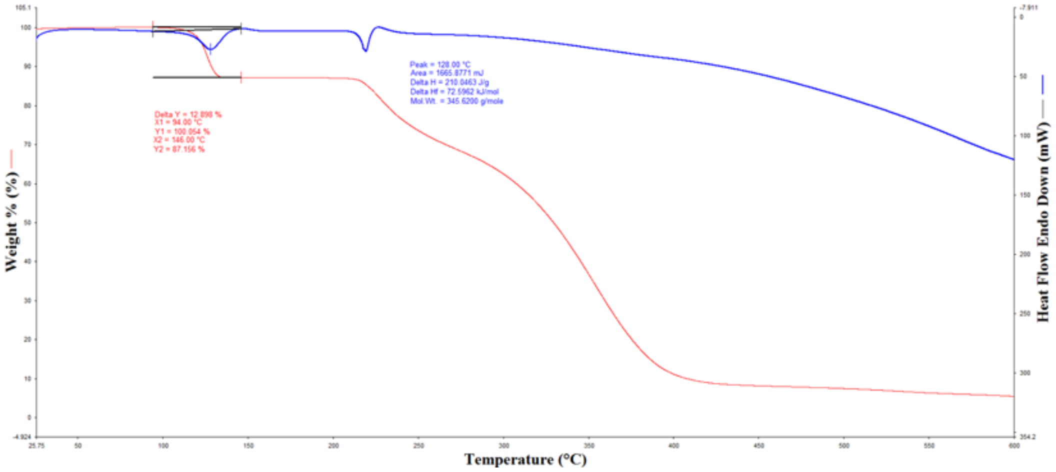

(b)

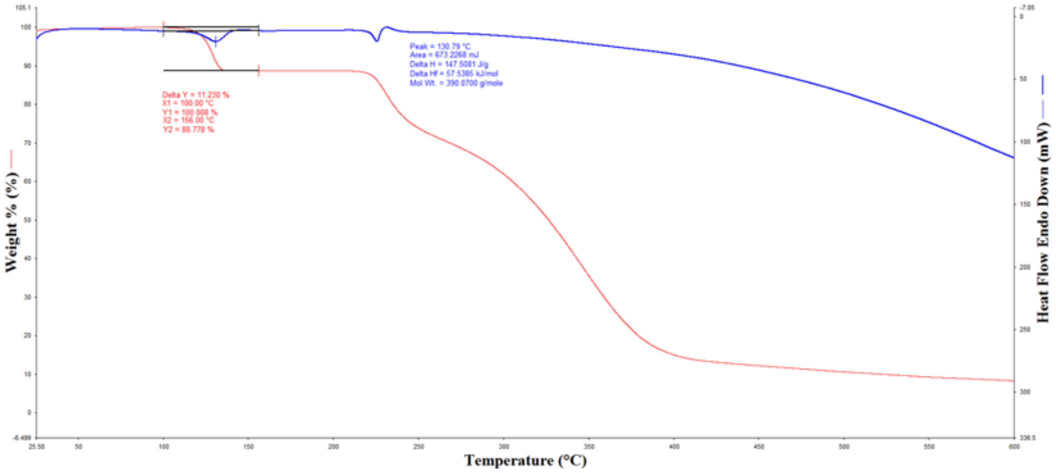

(c)

Figure S5. Traces for the STA conducted on (a) **1b**, (b) **1e** and (c) **1f**.

Data for the first weight loss (first exothermic process):

	Temp. range (°C)	Expt'l wght loss (%)	calcd wght loss (%)	$\Delta H_{desolvation}$ (kJ/mol)
**1b**.EtOH	90 – 162	88.8	86.6	46.7
**1e**.EtOH	94 – 146	87.2	86.7	72.6
**1f**.EtOH	100 – 156	88.8	88.2	57.5

Data for the second exothermic process:

	Temp. range (°C)	Peak temp. (°C)	$\Delta H_{melting}$ (kJ/mol)
**1e**.EtOH	204 – 227	219.4	29.2
**1f**.EtOH	211 – 232	225.9	27.6

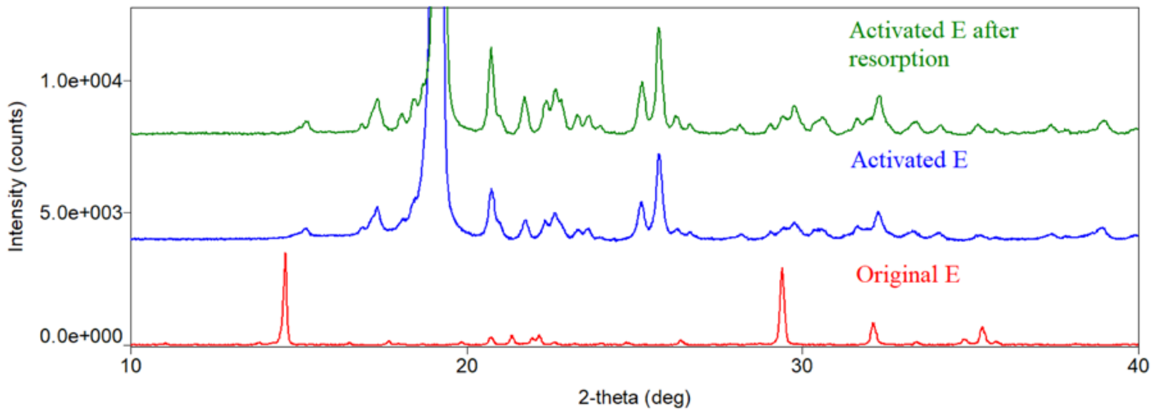

(a)

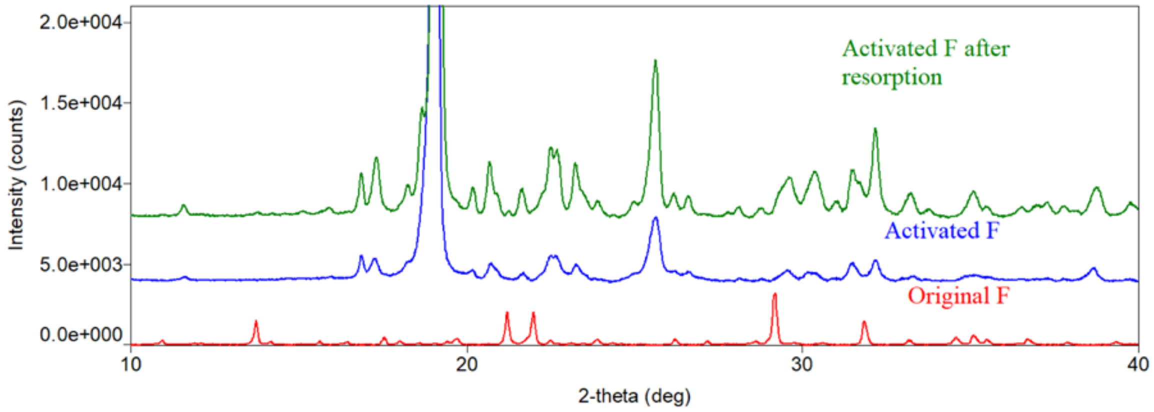

(b)

Figure S6. Experimental powder X-ray diffraction patterns for (a) **1e** and (b) **1f**. The red trace is of the original materials, *i.e.* **1e**.EtOH and **1f**.EtOH. The blue traces are of the desolvated materials as per the STA analyses, showing that the crystal structures are different. The green traces are measurements taken after exposing the desolvated materials to EtOH vapour overnight, showing no reversion to the original structures of **1e**.EtOH and **1f**.EtOH had occurred.

Copies of NMR spectra of prepared products **1a-k · EtOH** and **1a**

N²,6-Diphenyl-5,6-dihydro-1,3,5-triazine-2,4-diamine ethanolate (1a · EtOH)

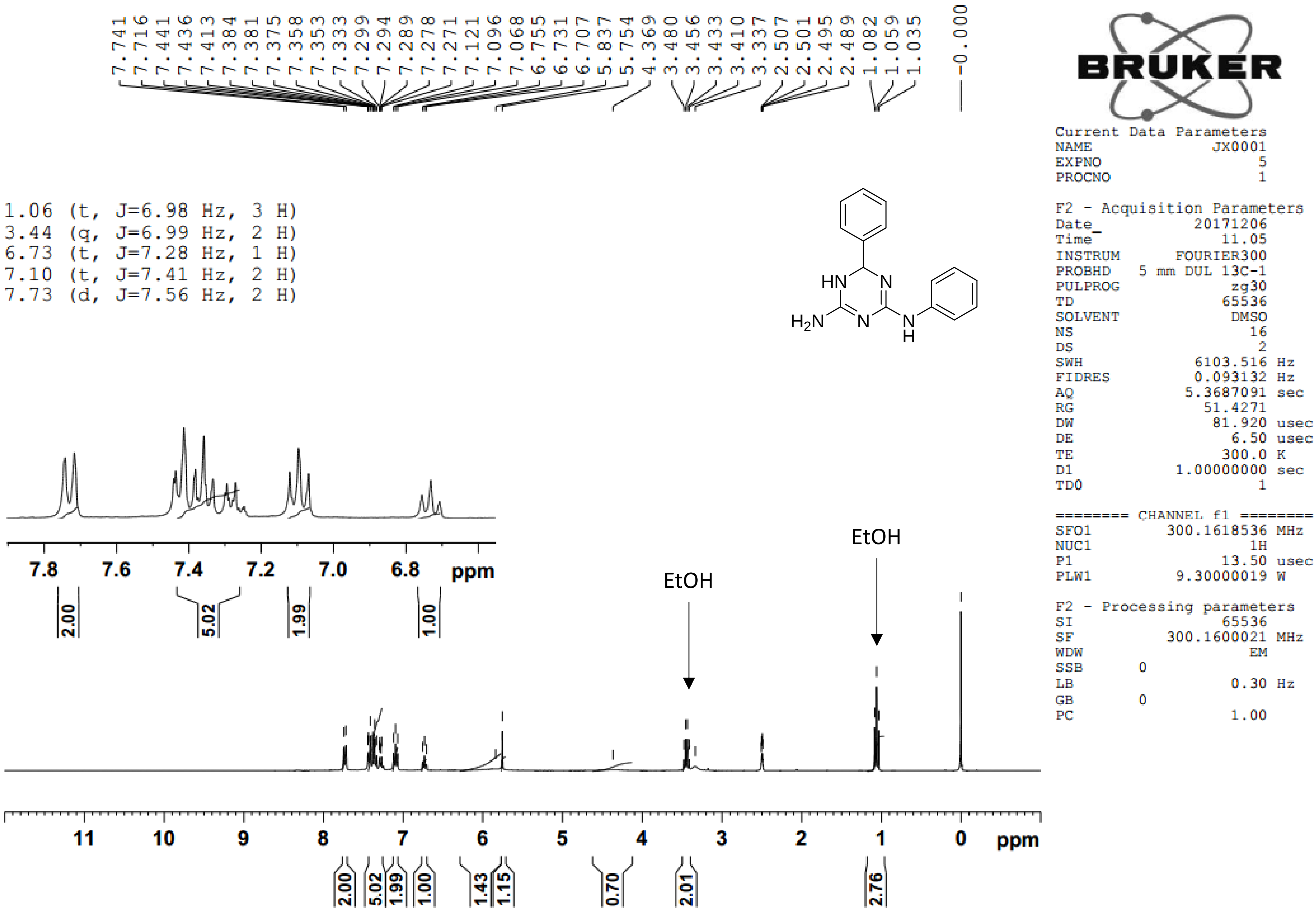

N[2],6-Diphenyl-5,6-dihydro-1,3,5-triazine-2,4-diamine ethanolate (1a · EtOH)

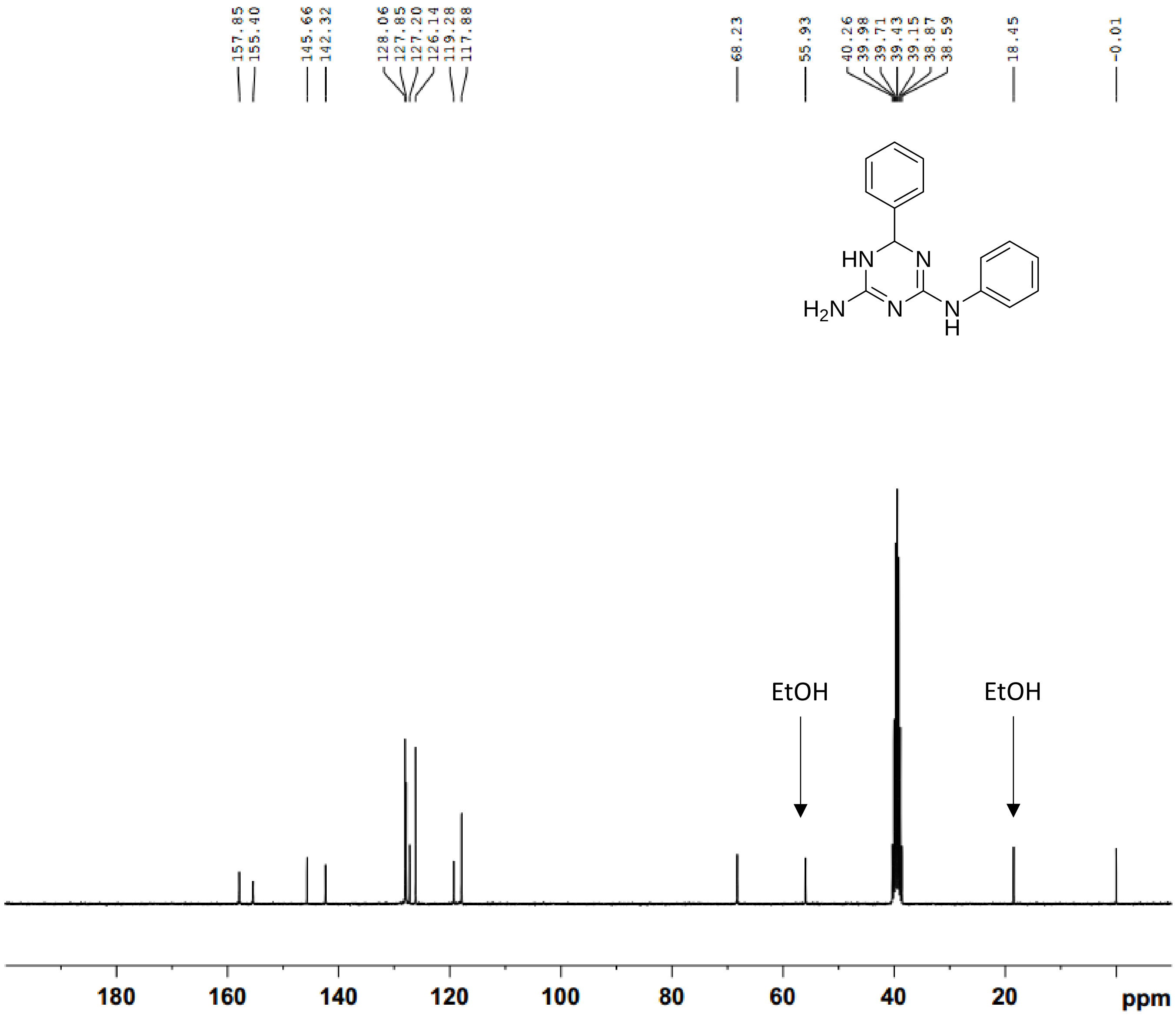


```
Current Data Parameters
NAME                E0036
EXPNO                   6
PROCNO                  1

F2 - Acquisition Parameters
Date_            20171206
Time                22.04
INSTRUM        FOURIER300
PROBHD    5 mm DUL 13C-1
PULPROG           zgpg30
TD                 65536
SOLVENT             DMSO
NS                  3072
DS                     4
SWH            24414.063 Hz
FIDRES          0.372529 Hz
AQ             1.3421773 sec
RG               501.187
DW                20.480 usec
DE                  6.50 usec
TE                 300.2 K
D1            2.00000000 sec
D11           0.03000000 sec
D31           0.00001500 sec
D40           0.00439029 sec
L4                    37
L5                    53
P32                98.00 usec
TD0                    3

======== CHANNEL f1 ========
SFO1          75.4828392 MHz
NUC1                 13C
P1                 15.00 usec
PLW1         22.00000000 W

======== CHANNEL f2 ========
SFO2         300.1612006 MHz
NUC2                  1H
CPDPRG[2         waltz16
PCPD2              98.00 usec
PLW2          9.30000019 W
PLW12         0.29359001 W
PLW13         0.20359001 W

F2 - Processing parameters
SI                 32768
SF            75.4753350 MHz
WDW                   EM
SSB         0
LB                  1.00 Hz
GB          0
PC                  1.40
```

N^2-(4-Chlorophenyl)-6-(4-methoxyphenyl)-5,6-dihydro-1,3,5-triazine-2,4-diamine ethanolate (1b · EtOH)

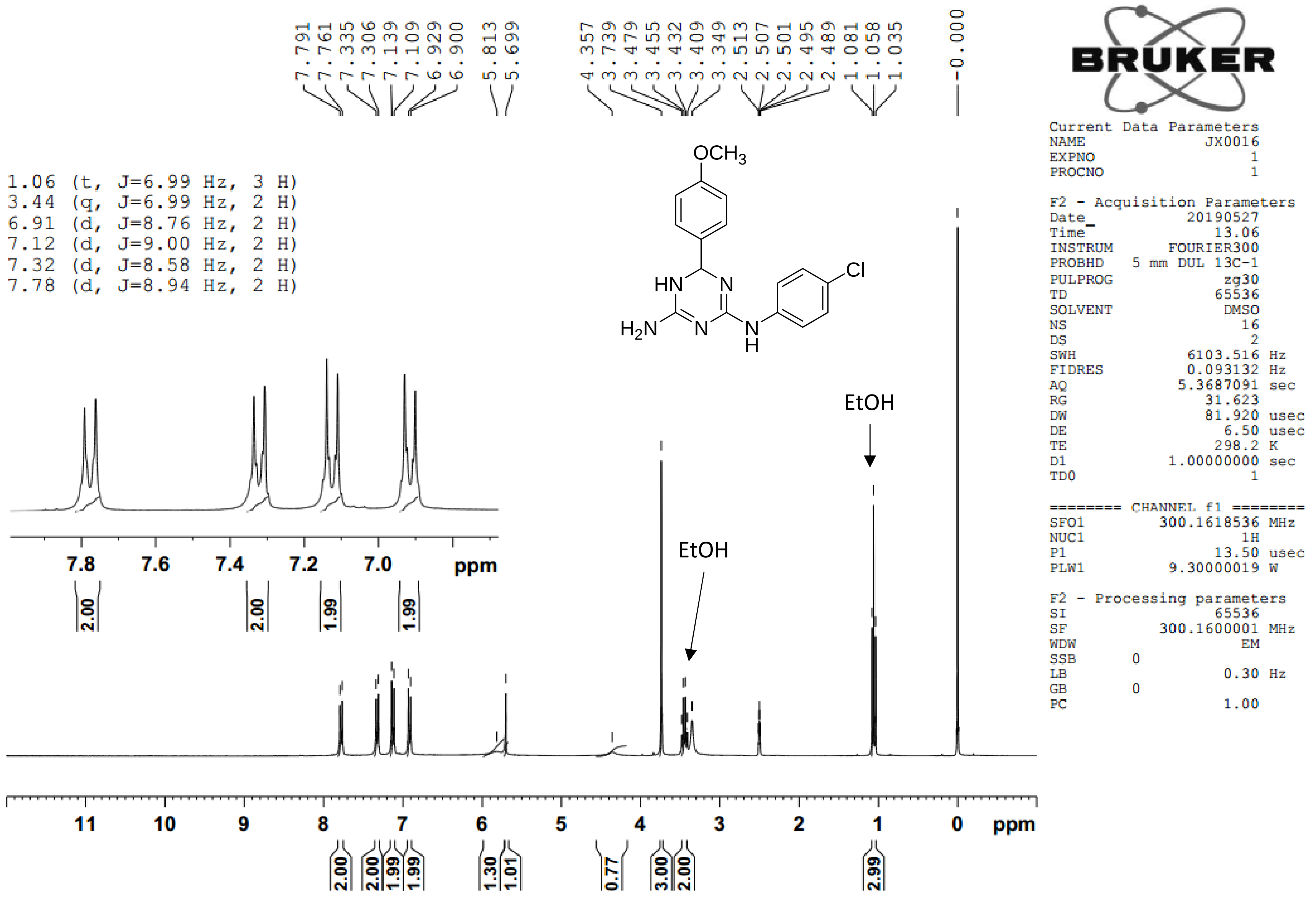

N*[2]-(4-Chlorophenyl)-6-(4-methoxyphenyl)-5,6-dihydro-1,3,5-triazine-2,4-diamine ethanolate (1b · EtOH)

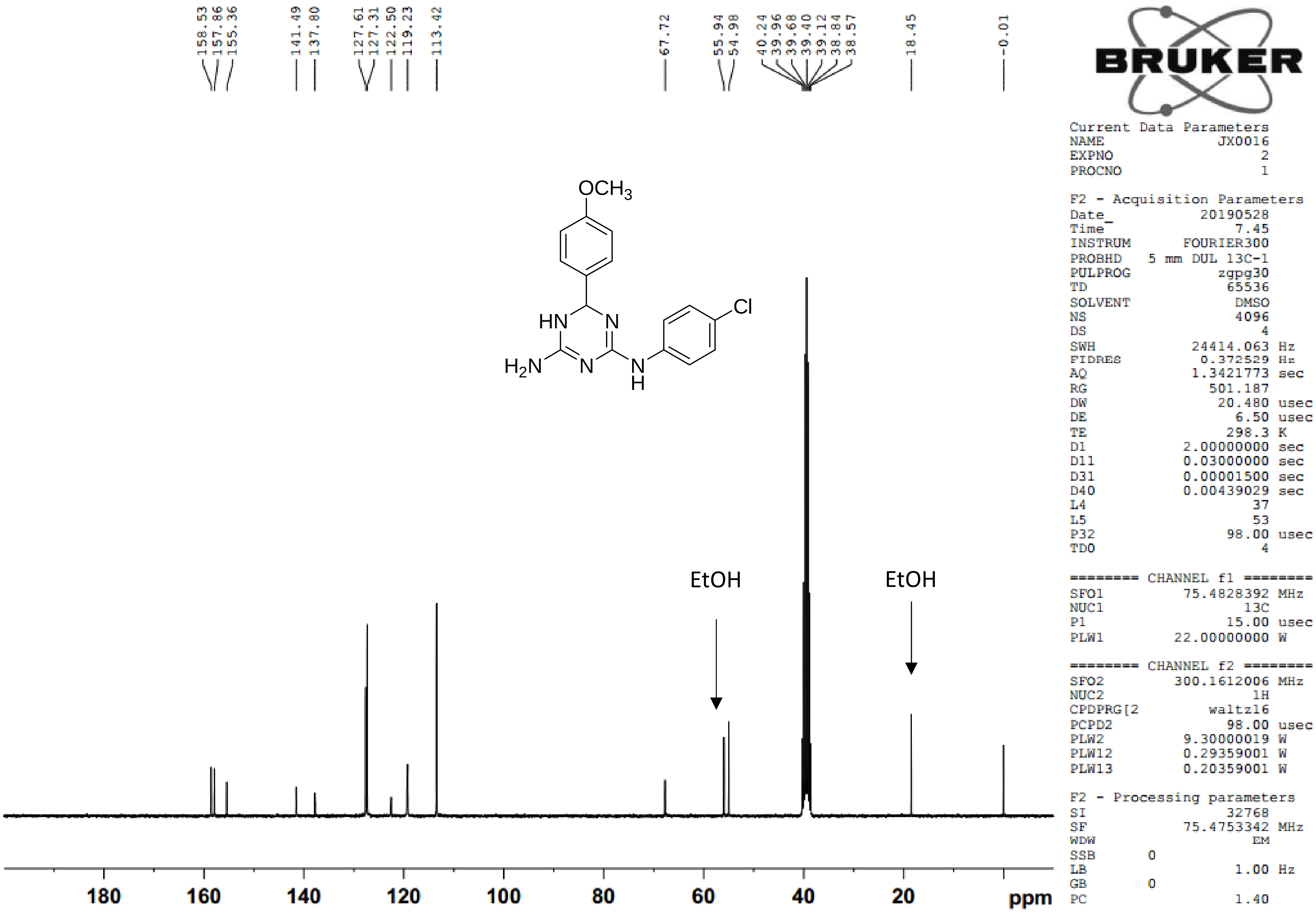

N²-(4-Fluorophenyl)-6-phenyl-5,6-dihydro-1,3,5-triazine-2,4-diamine ethanolate (1c · EtOH)

1.06 (t, J=6.99 Hz, 3 H)
3.45 (q, J=6.99 Hz, 2 H)
6.92 (dd, J=9.00, 9.00 Hz, 2 H)
7.76 (dd, J=5.12, 9.14 Hz, 2 H)

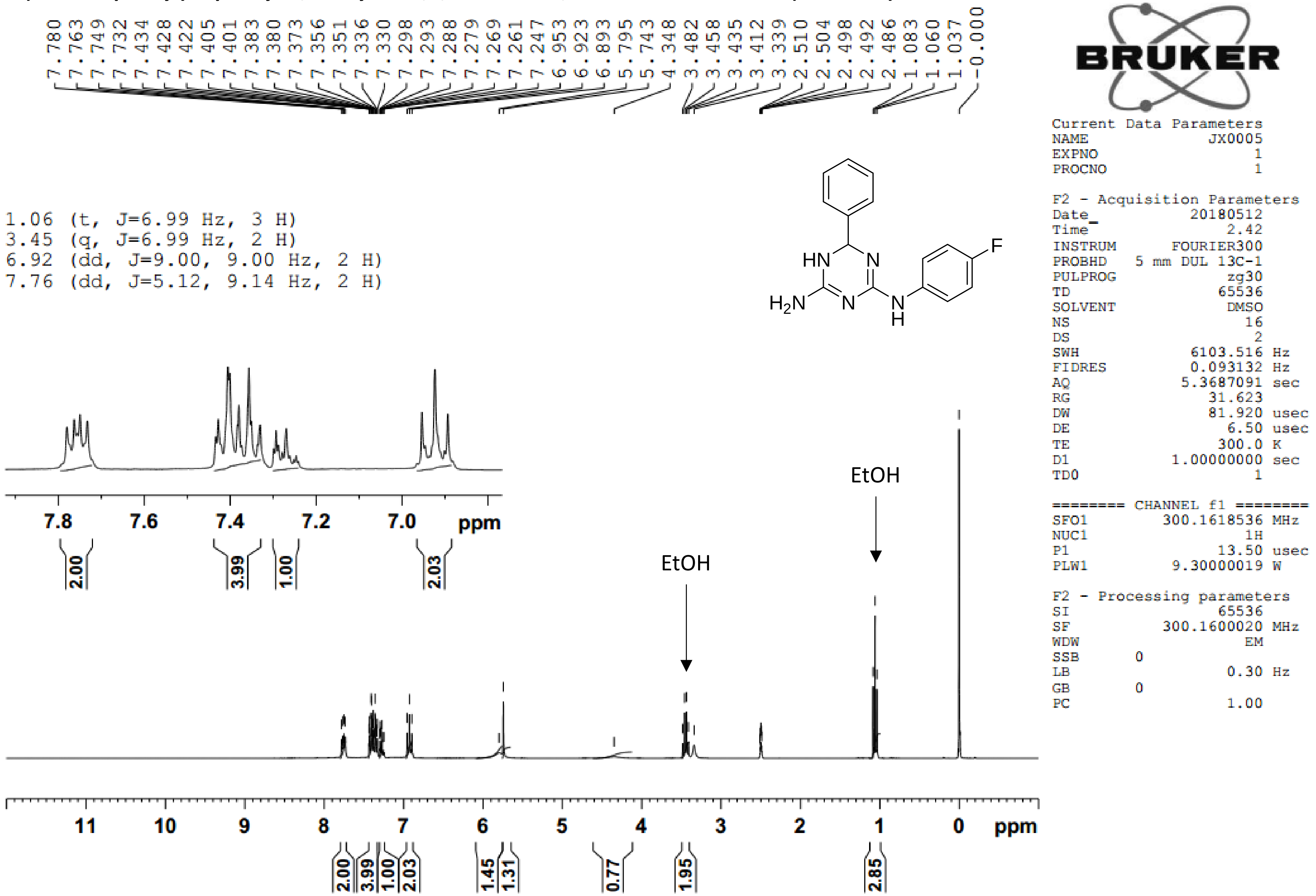

N[2]-(4-Fluorophenyl)-6-phenyl-5,6-dihydro-1,3,5-triazine-2,4-diamine ethanolate (1c · EtOH)

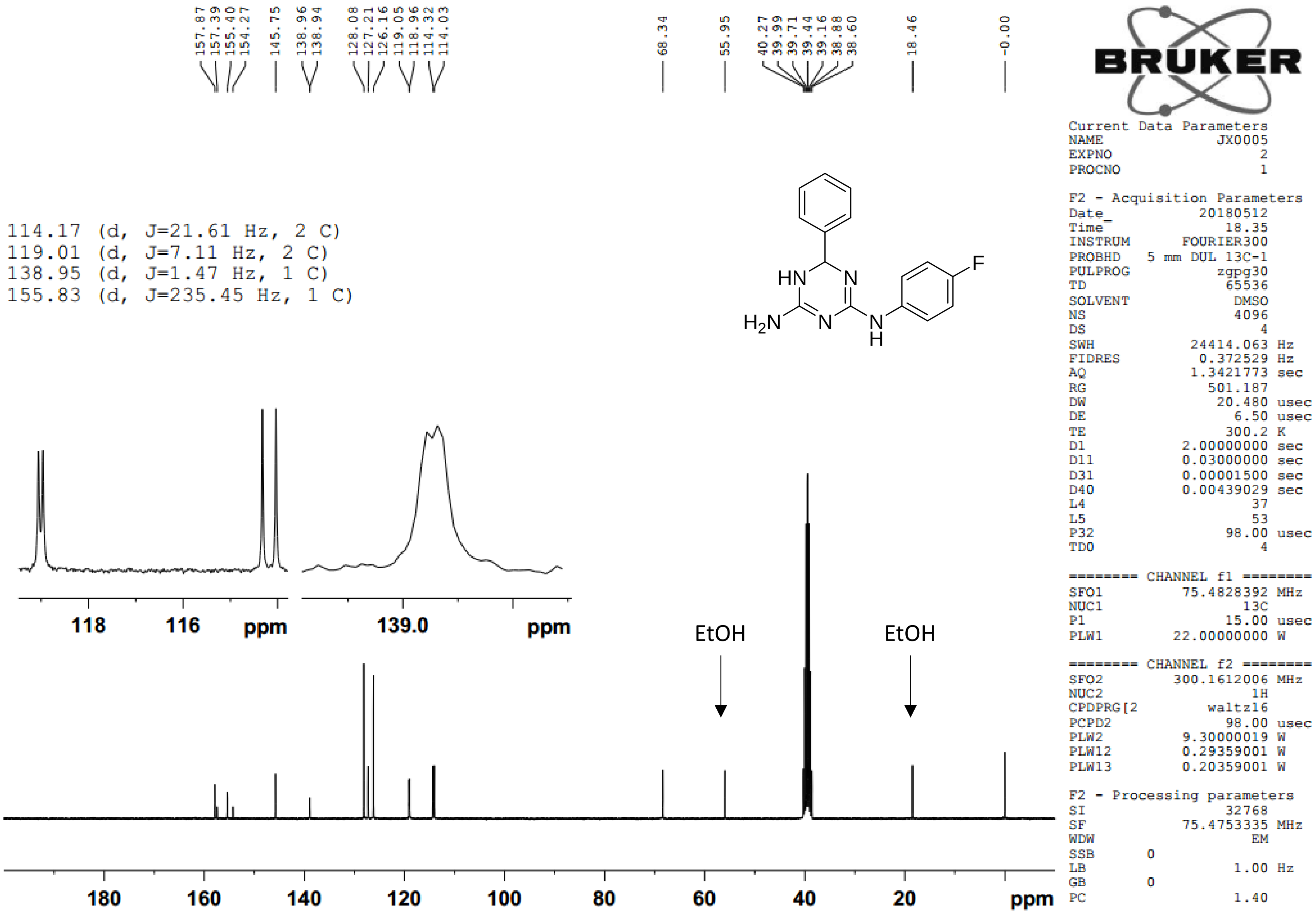

N[2]-(3-Chlorophenyl)-6-phenyl-5,6-dihydro-1,3,5-triazine-2,4-diamine ethanolate (1d · EtOH)

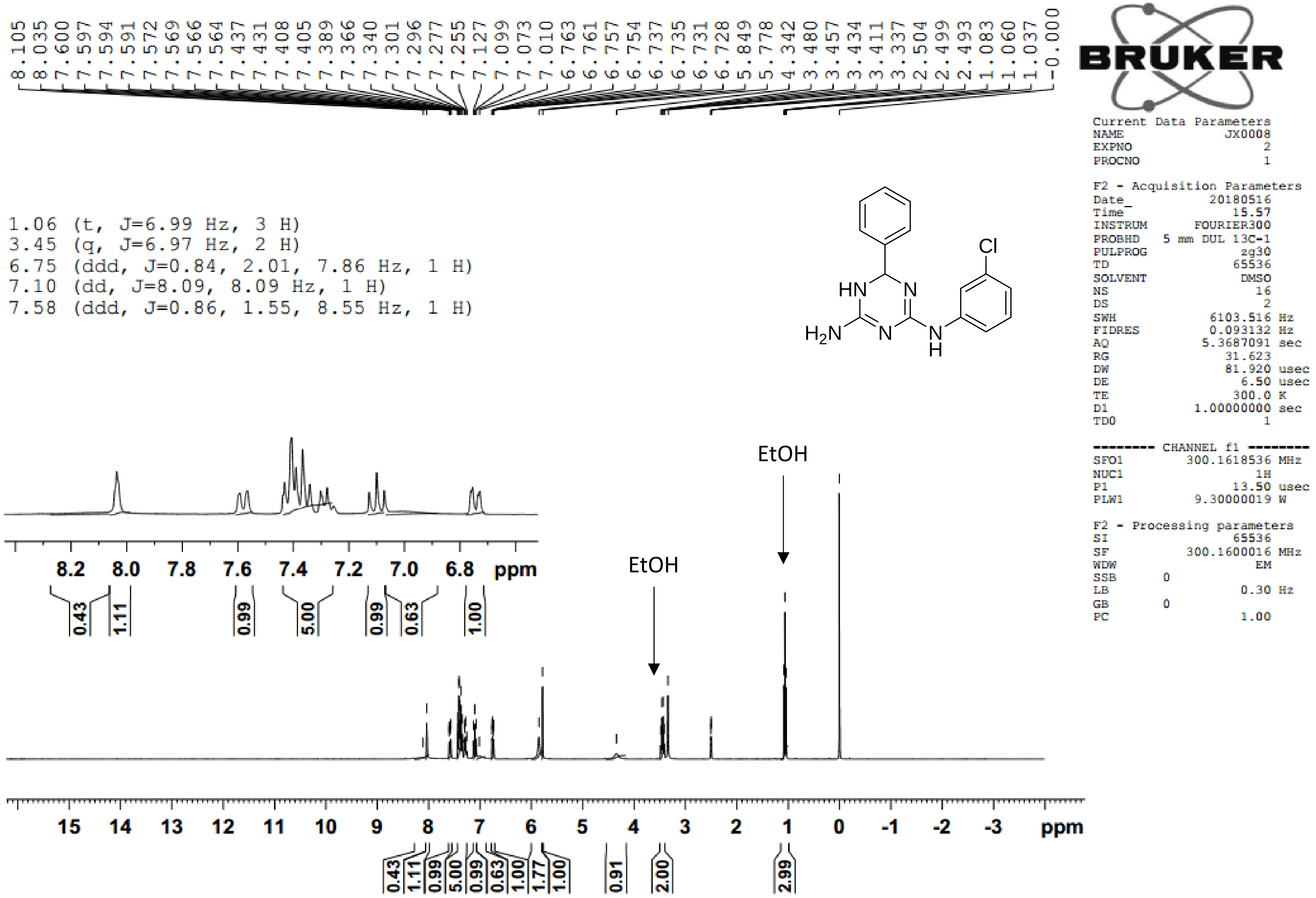

N[2]-(3-Chlorophenyl)-6-phenyl-5,6-dihydro-1,3,5-triazine-2,4-diamine ethanolate (1d · EtOH)*

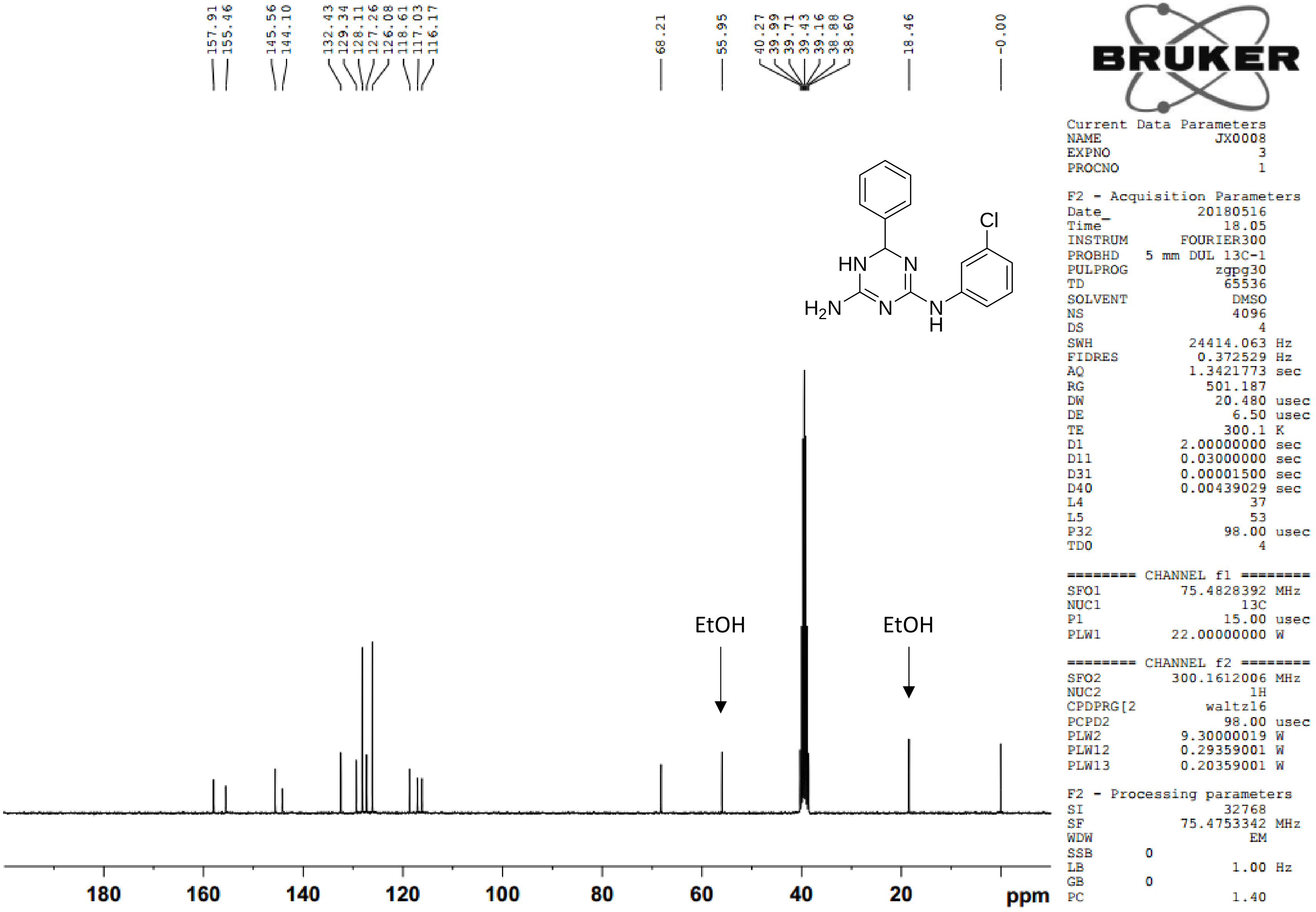

N^2-(4-Chlorophenyl)-6-phenyl-5,6-dihydro-1,3,5-triazine-2,4-diamine ethanolate (1e · EtOH)

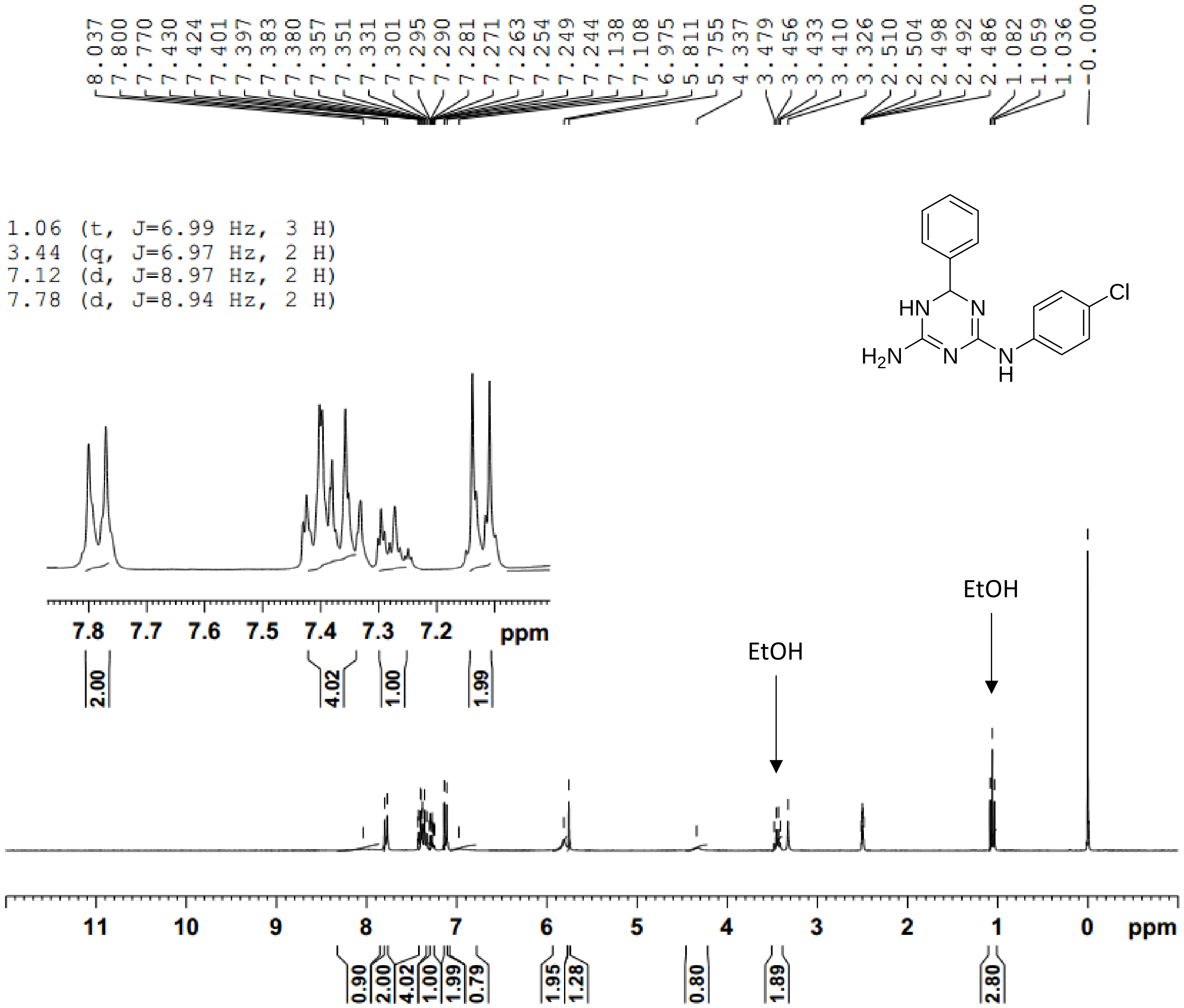

BRUKER

```
Current Data Parameters
NAME                 JX0003
EXPNO                     2
PROCNO                    1

F2 - Acquisition Parameters
Date_              20180404
Time                  13.19
INSTRUM          FOURIER300
PROBHD     5 mm DUL 13C-1
PULPROG                zg30
TD                    65536
SOLVENT                DMSO
NS                       16
DS                        2
SWH                6103.516 Hz
FIDRES             0.093132 Hz
AQ                5.3687091 sec
RG                  60.5742
DW                   81.920 usec
DE                     6.50 usec
TE                    300.1 K
D1               1.00000000 sec
TD0                       1

======== CHANNEL f1 ========
SFO1            300.1618536 MHz
NUC1                     1H
P1                    13.50 usec
PLW1             9.30000019 W

F2 - Processing parameters
SI                    65536
SF              300.1600016 MHz
WDW                      EM
SSB      0
LB                     0.30 Hz
GB       0
PC                     1.00
```

***N*²-(4-Chlorophenyl)-6-phenyl-5,6-dihydro-1,3,5-triazine-2,4-diamine ethanolate (1e · EtOH)**

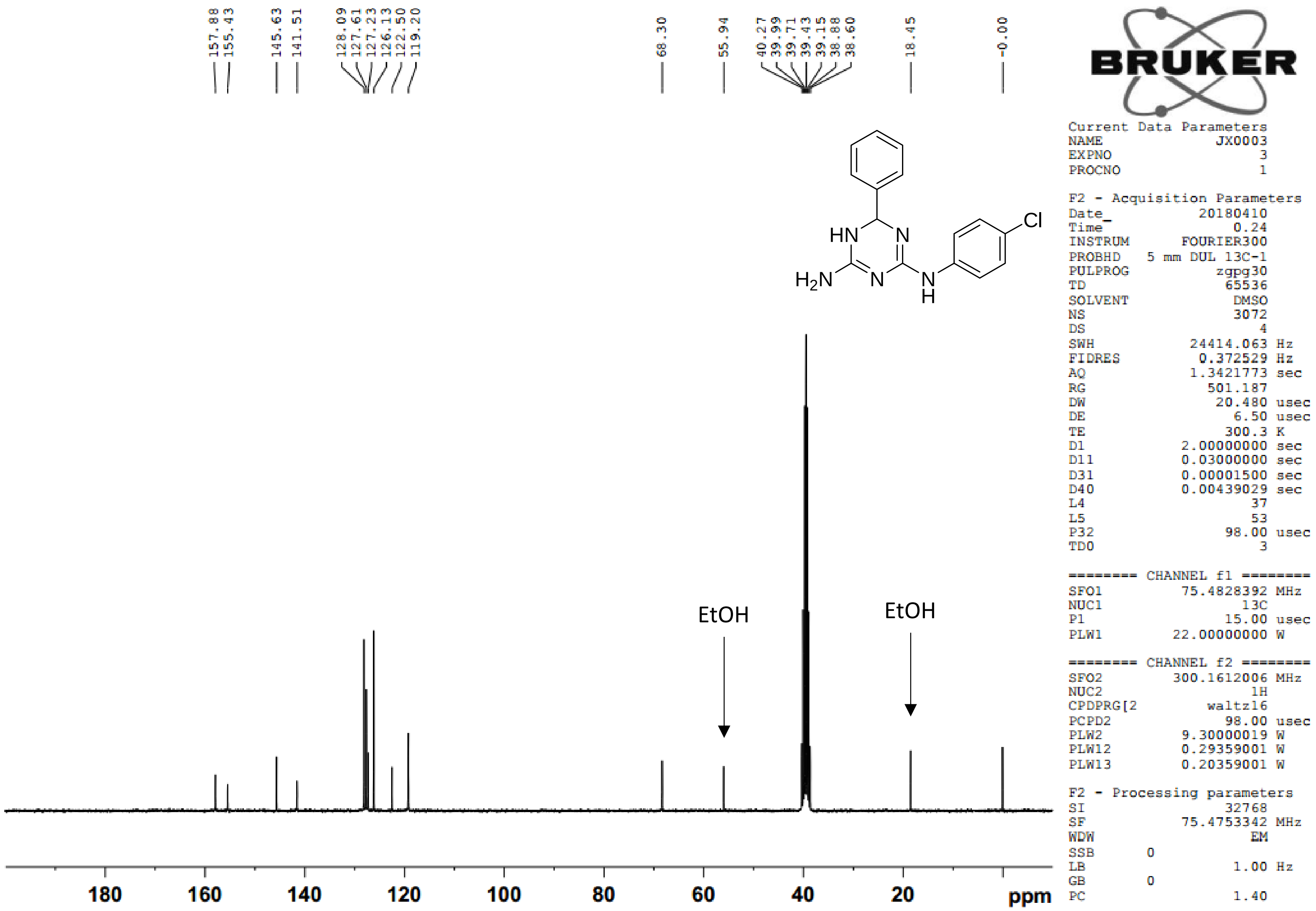

N*[2]-(4-Bromophenyl)-6-phenyl-5,6-dihydro-1,3,5-triazine-2,4-diamine ethanolate (1f · EtOH)

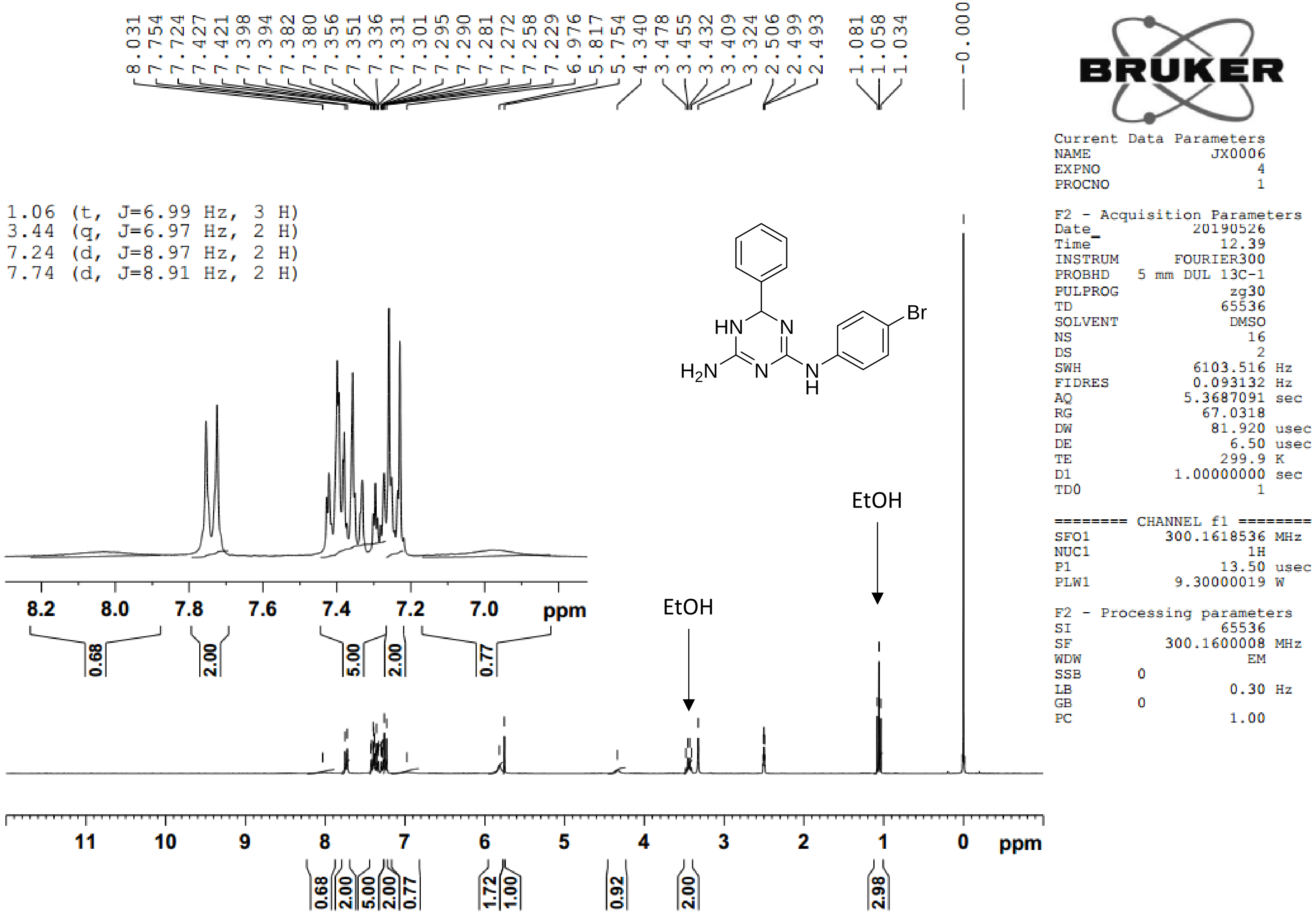

N[2]-(4-Bromophenyl)-6-phenyl-5,6-dihydro-1,3,5-triazine-2,4-diamine ethanolate (1f · EtOH)

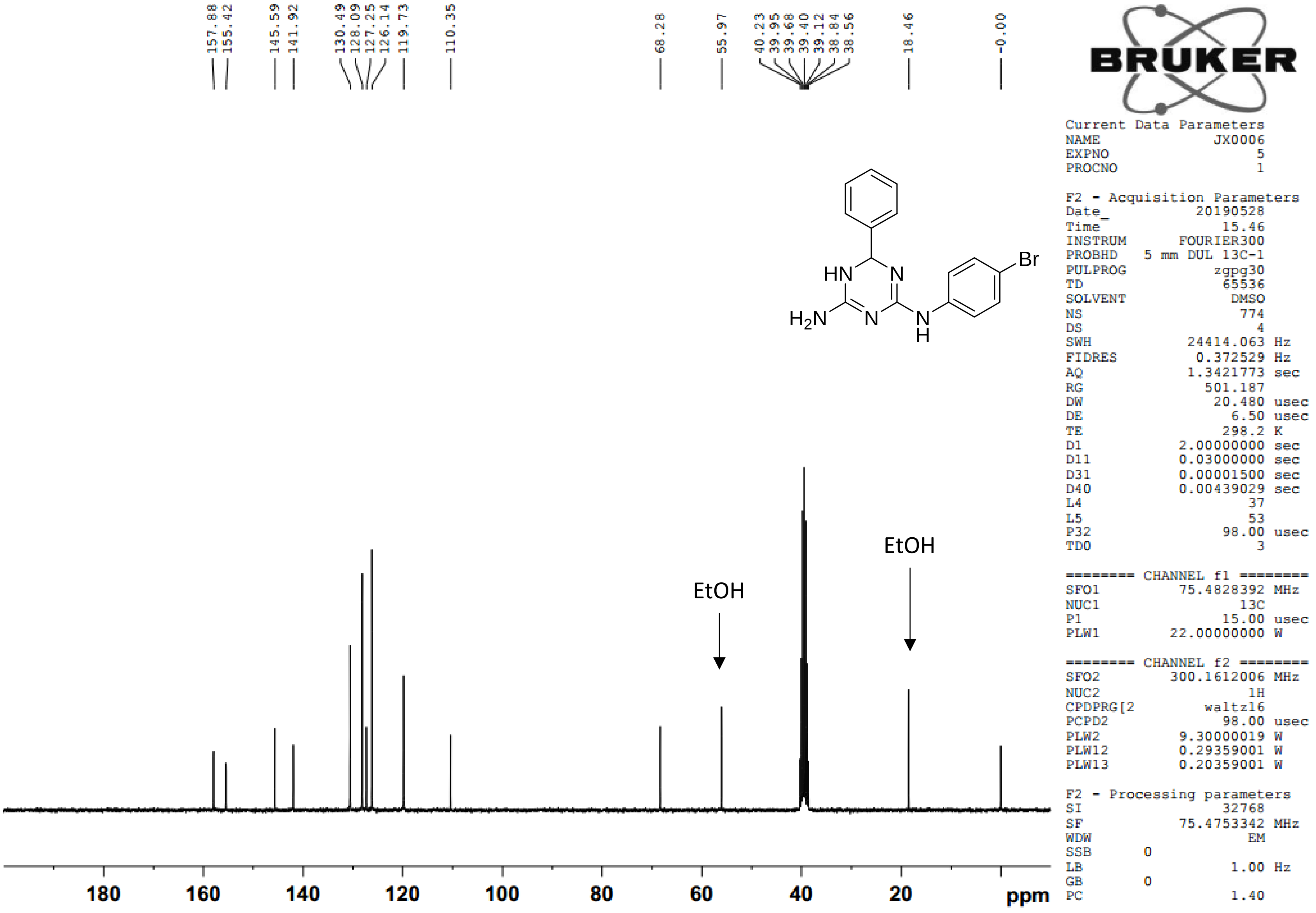

N*[2]-(4-Methoxyphenyl)-6-phenyl-5,6-dihydro-1,3,5-triazine-2,4-diamine ethanolate (1g · EtOH)

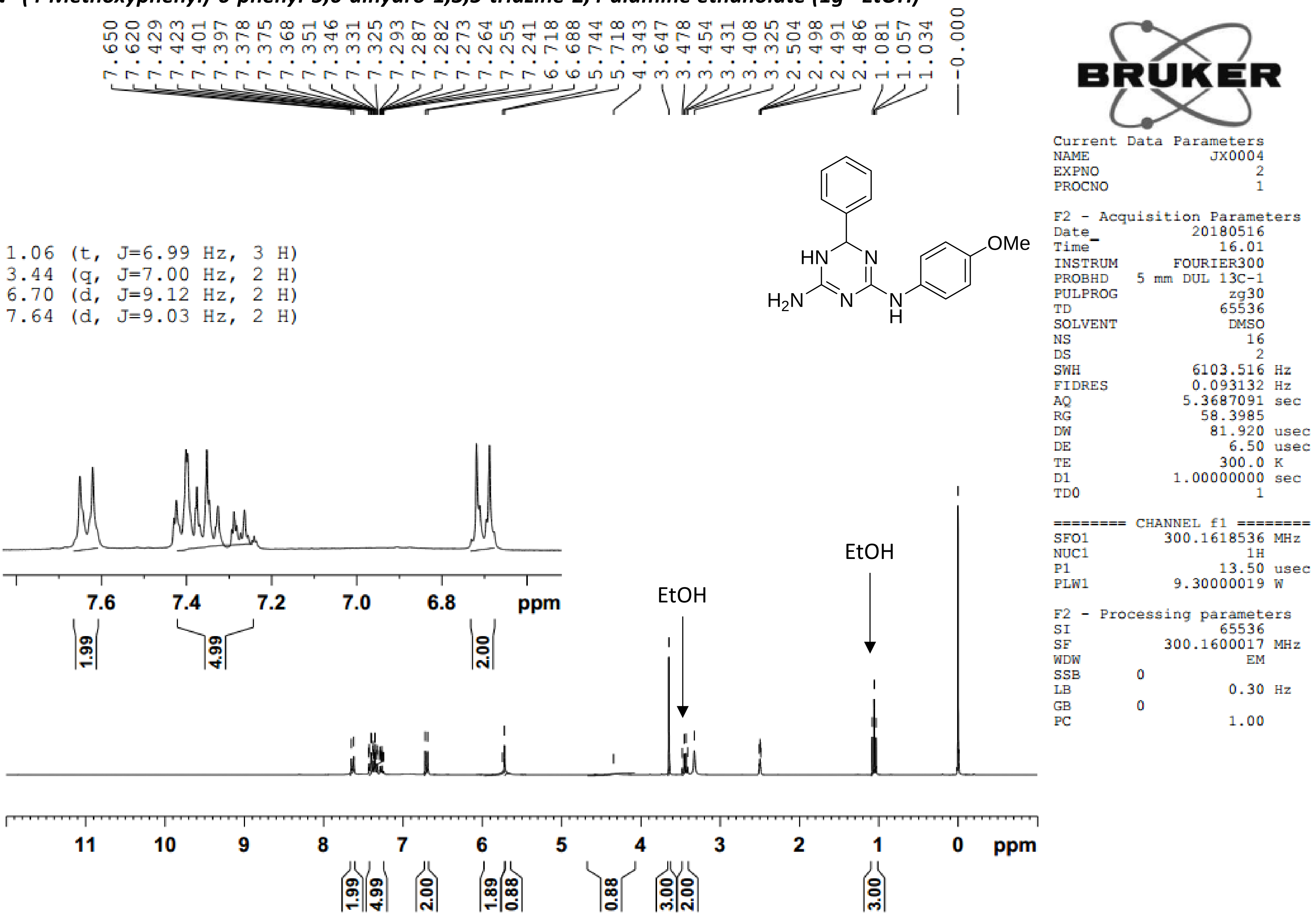

N[2]-(4-Methoxyphenyl)-6-phenyl-5,6-dihydro-1,3,5-triazine-2,4-diamine ethanolate (1g · EtOH)

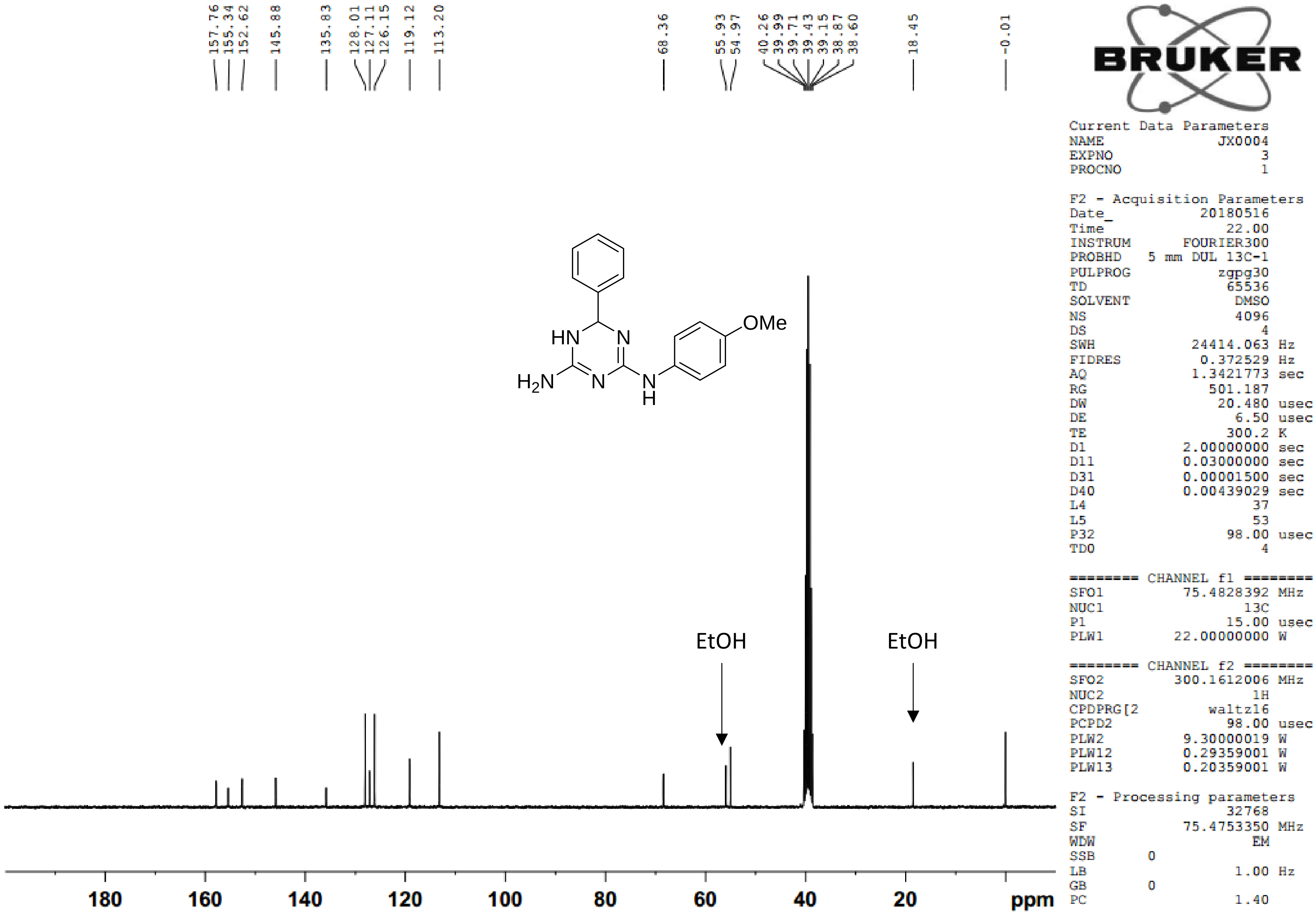

N^2-(3-Methylphenyl)-6-phenyl-5,6-dihydro-1,3,5-triazine-2,4-diamine ethanolate (1h · EtOH)

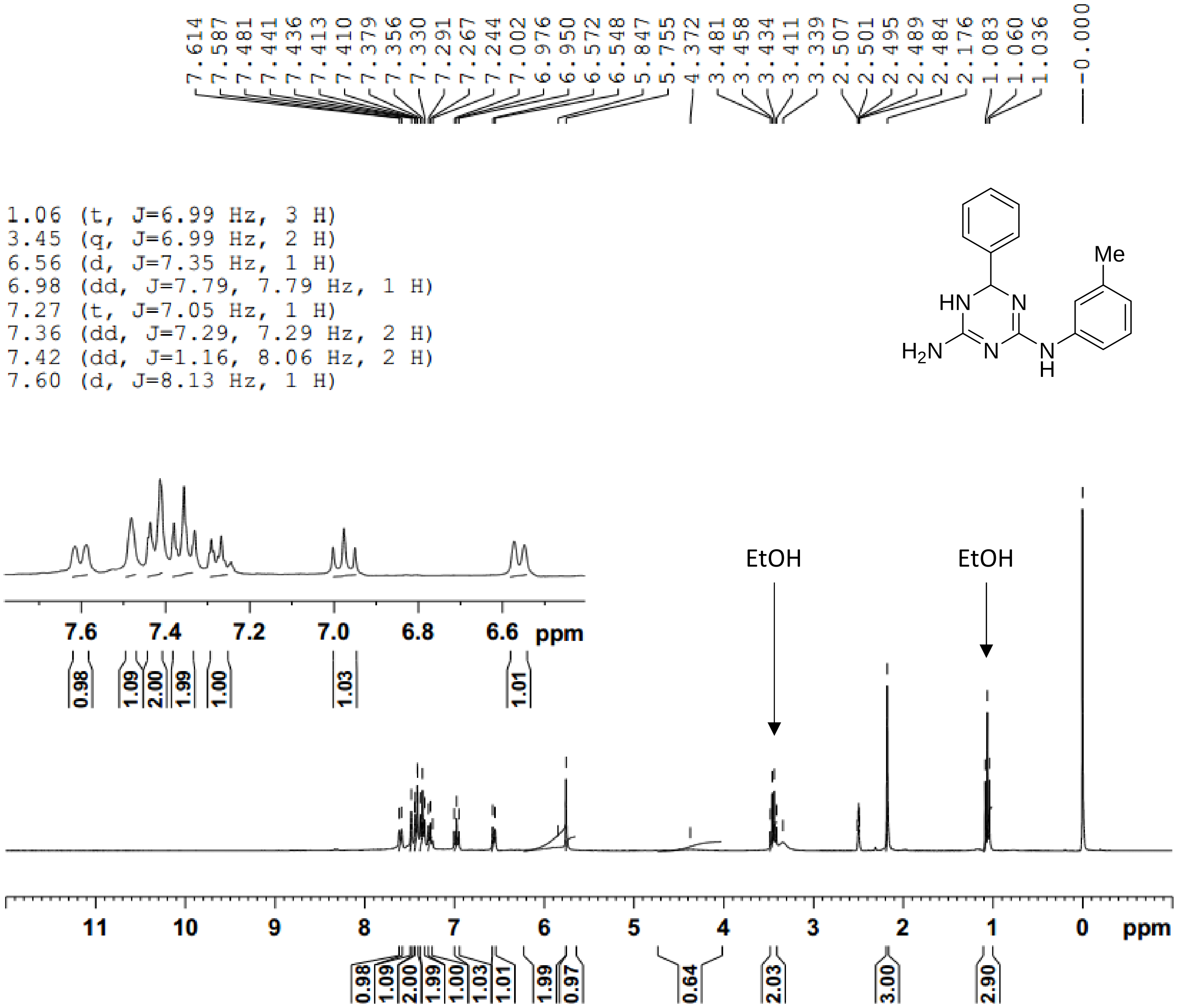

BRUKER

Current Data Parameters
NAME JX0007
EXPNO 1
PROCNO 1

F2 - Acquisition Parameters
Date_ 20180513
Time 14.36
INSTRUM FOURIER300
PROBHD 5 mm DUL 13C-1
PULPROG zg30
TD 65536
SOLVENT DMSO
NS 16
DS 2
SWH 6103.516 Hz
FIDRES 0.093132 Hz
AQ 5.3687091 sec
RG 31.623
DW 81.920 usec
DE 6.50 usec
TE 300.0 K
D1 1.00000000 sec
TD0 1

======== CHANNEL f1 ========
SFO1 300.1618536 MHz
NUC1 1H
P1 13.50 usec
PLW1 9.30000019 W

F2 - Processing parameters
SI 65536
SF 300.1600025 MHz
WDW EM
SSB 0
LB 0.30 Hz
GB 0
PC 1.00

***N*[2]-(3-Methylphenyl)-6-phenyl-5,6-dihydro-1,3,5-triazine-2,4-diamine ethanolate (1h · EtOH)**

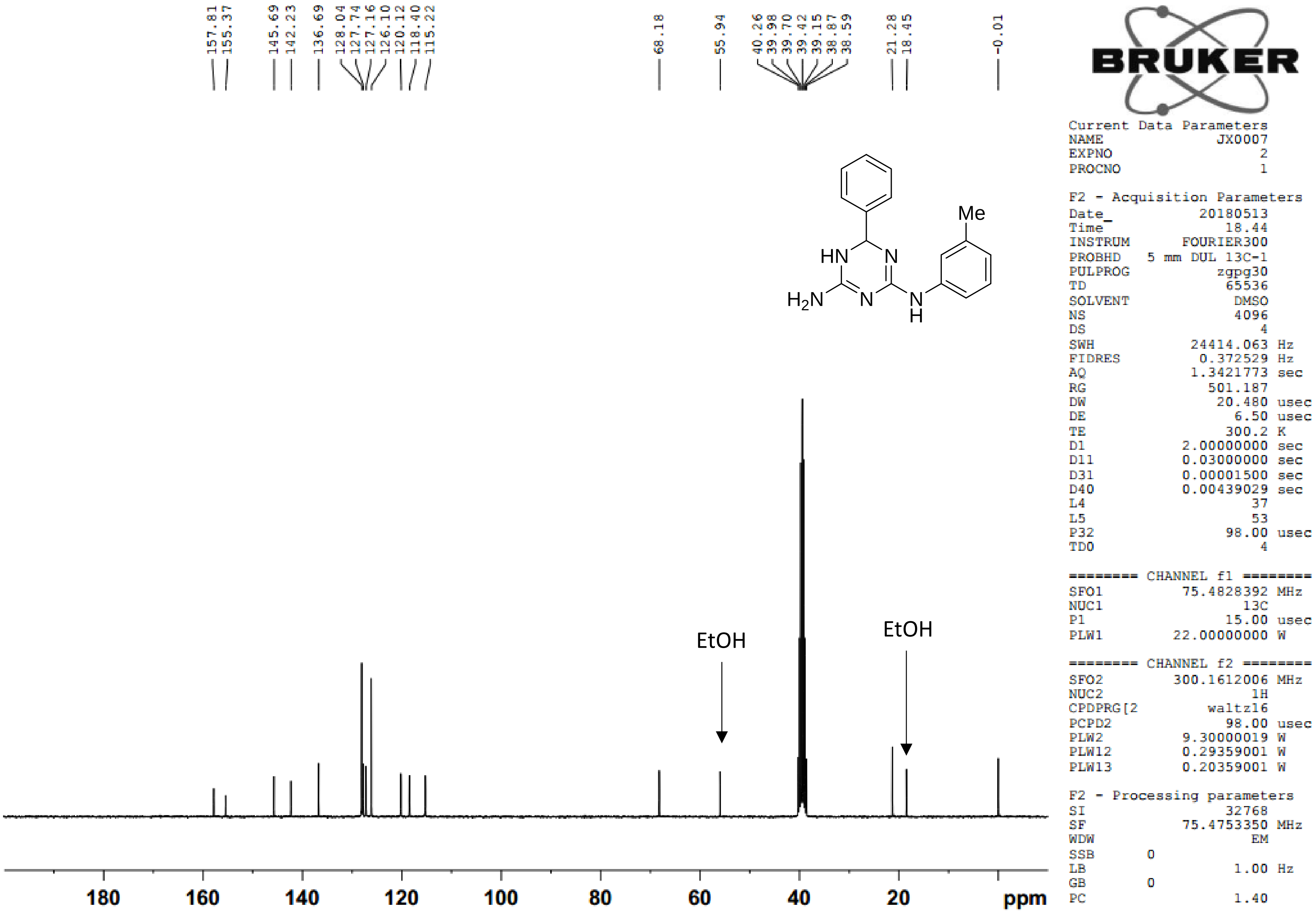

N^2-(4-Methylphenyl)-6-phenyl-5,6-dihydro-1,3,5-triazine-2,4-diamine ethanolate (1i · EtOH)

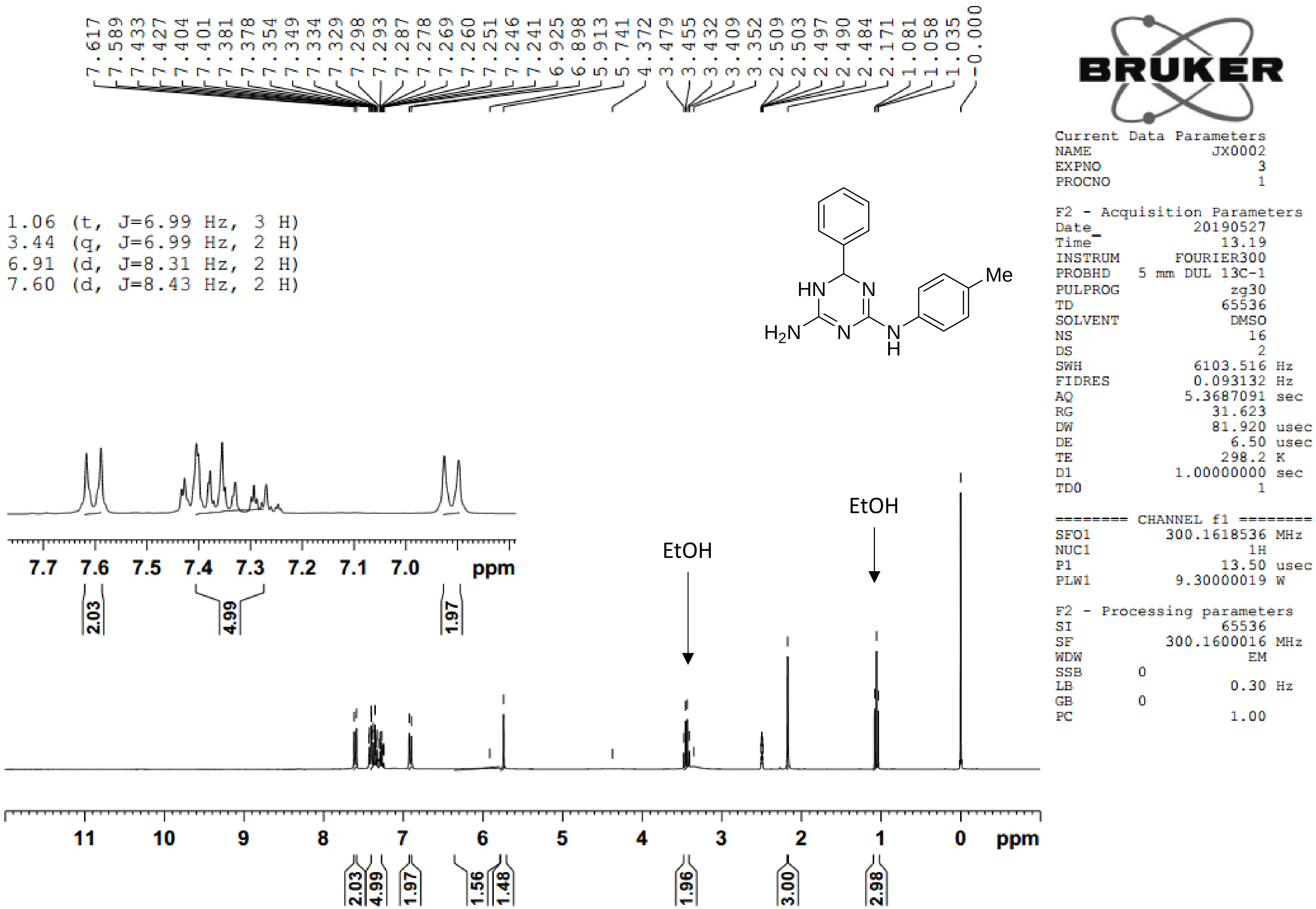

***N*[2]-(4-Methylphenyl)-6-phenyl-5,6-dihydro-1,3,5-triazine-2,4-diamine ethanolate (1i · EtOH)**

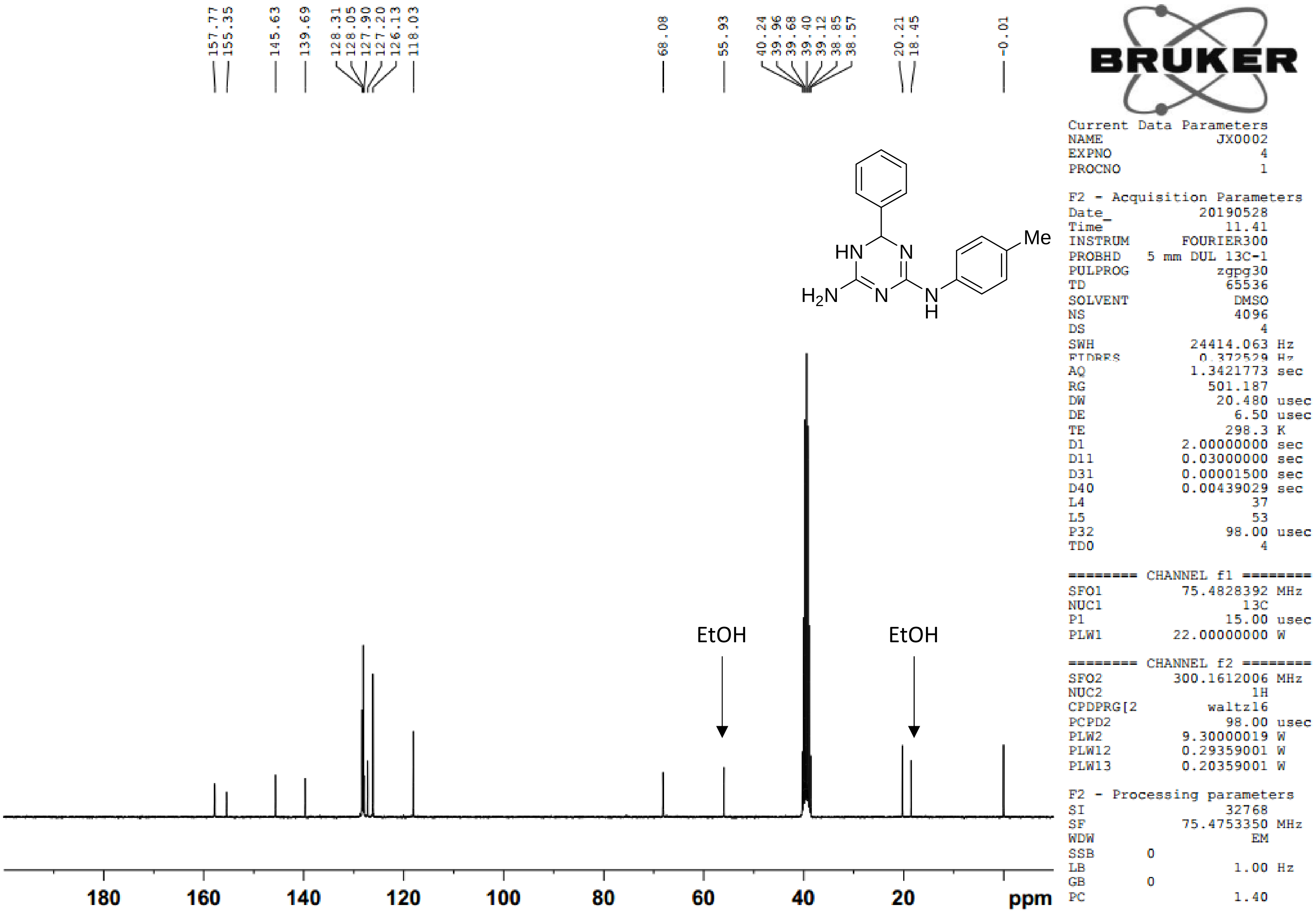

6-Phenyl-N²-(4-(trifluoromethoxy)phenyl)-5,6-dihydro-1,3,5-triazine-2,4-diamine ethanolate (1j · EtOH)

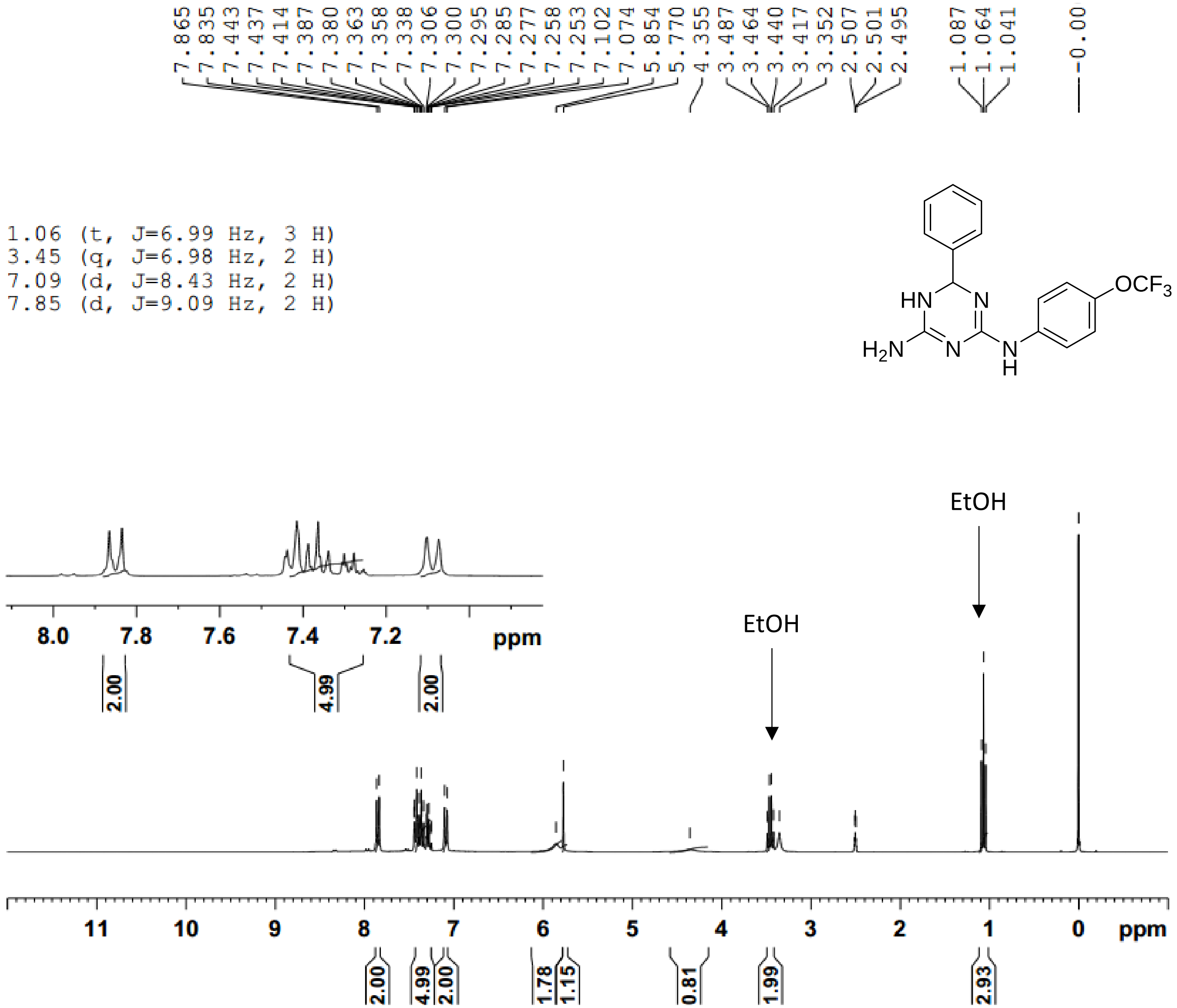

Current Data Parameters
NAME JX0010
EXPNO 1
PROCNO 1

F2 - Acquisition Parameters
Date_ 20180801
Time 16.05
INSTRUM FOURIER300
PROBHD 5 mm DUL 13C-1
PULPROG zg30
TD 65536
SOLVENT DMSO
NS 16
DS 2
SWH 6103.516 Hz
FIDRES 0.093132 Hz
AQ 5.3687091 sec
RG 31.623
DW 81.920 usec
DE 6.50 usec
TE 300.2 K
D1 1.00000000 sec
TD0 1

======== CHANNEL f1 ========
SFO1 300.1618536 MHz
NUC1 1H
P1 13.50 usec
PLW1 9.30000019 W

F2 - Processing parameters
SI 65536
SF 300.1600002 MHz
WDW EM
SSB 0
LB 0.30 Hz
GB 0
PC 1.00

6-Phenyl-N²-(4-(trifluoromethoxy)phenyl)-5,6-dihydro-1,3,5-triazine-2,4-diamine ethanolate (1j · EtOH)

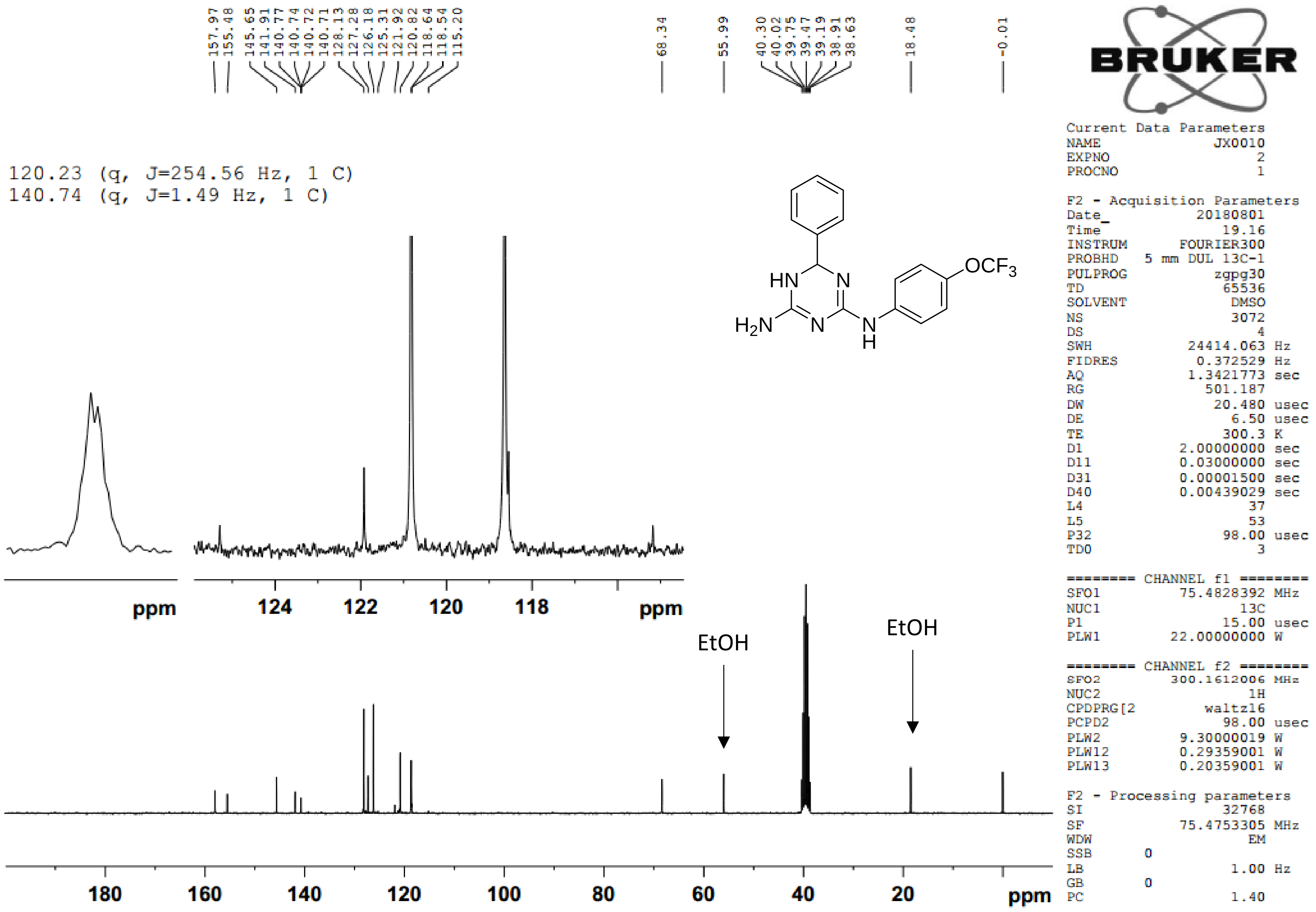

6-(4-Chlorophenyl)-N^2-phenyl-5,6-dihydro-1,3,5-triazine-2,4-diamine ethanolate (1k · EtOH)

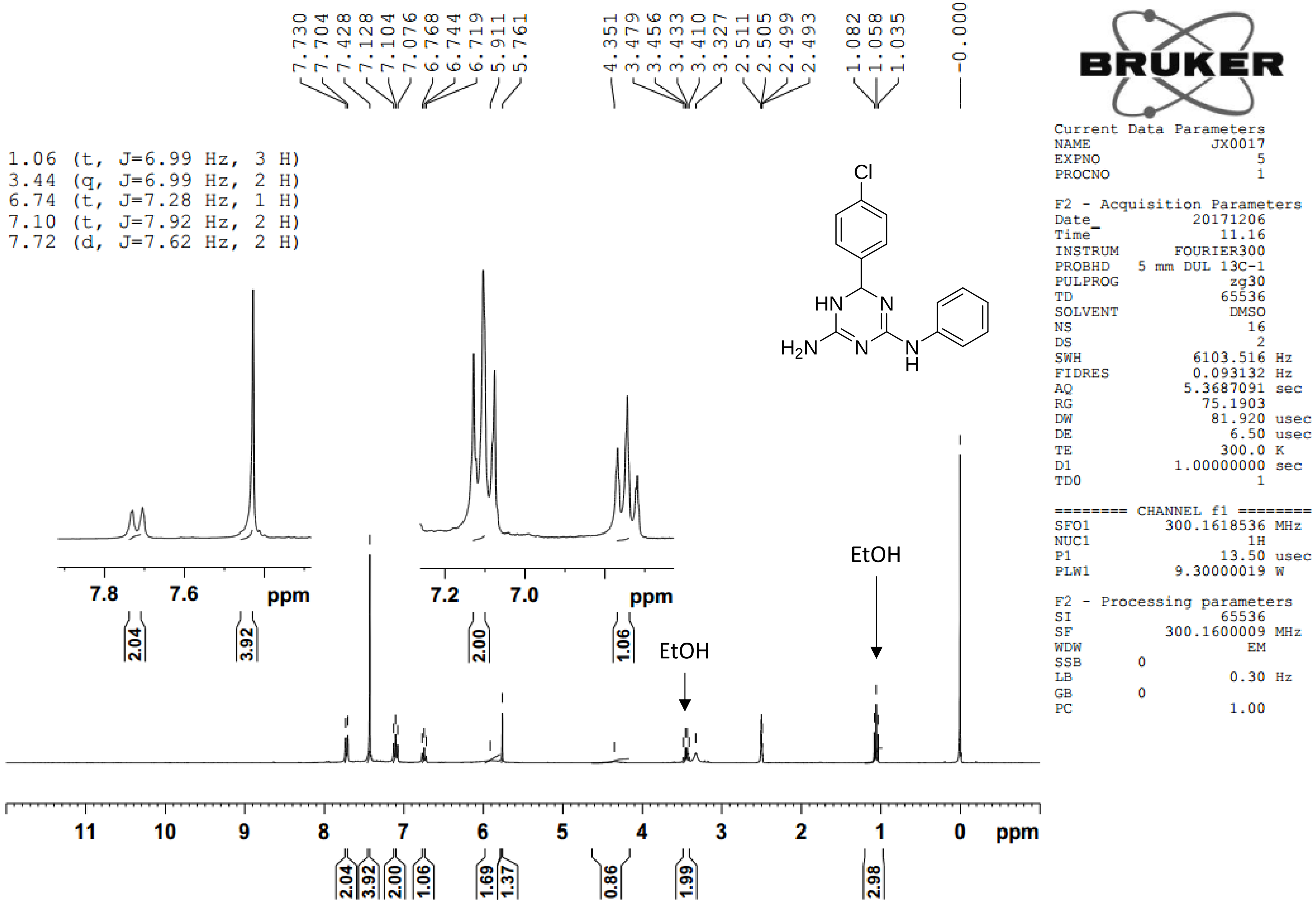

6-(4-Chlorophenyl)-N²-phenyl-5,6-dihydro-1,3,5-triazine-2,4-diamine ethanolate (1k · EtOH)

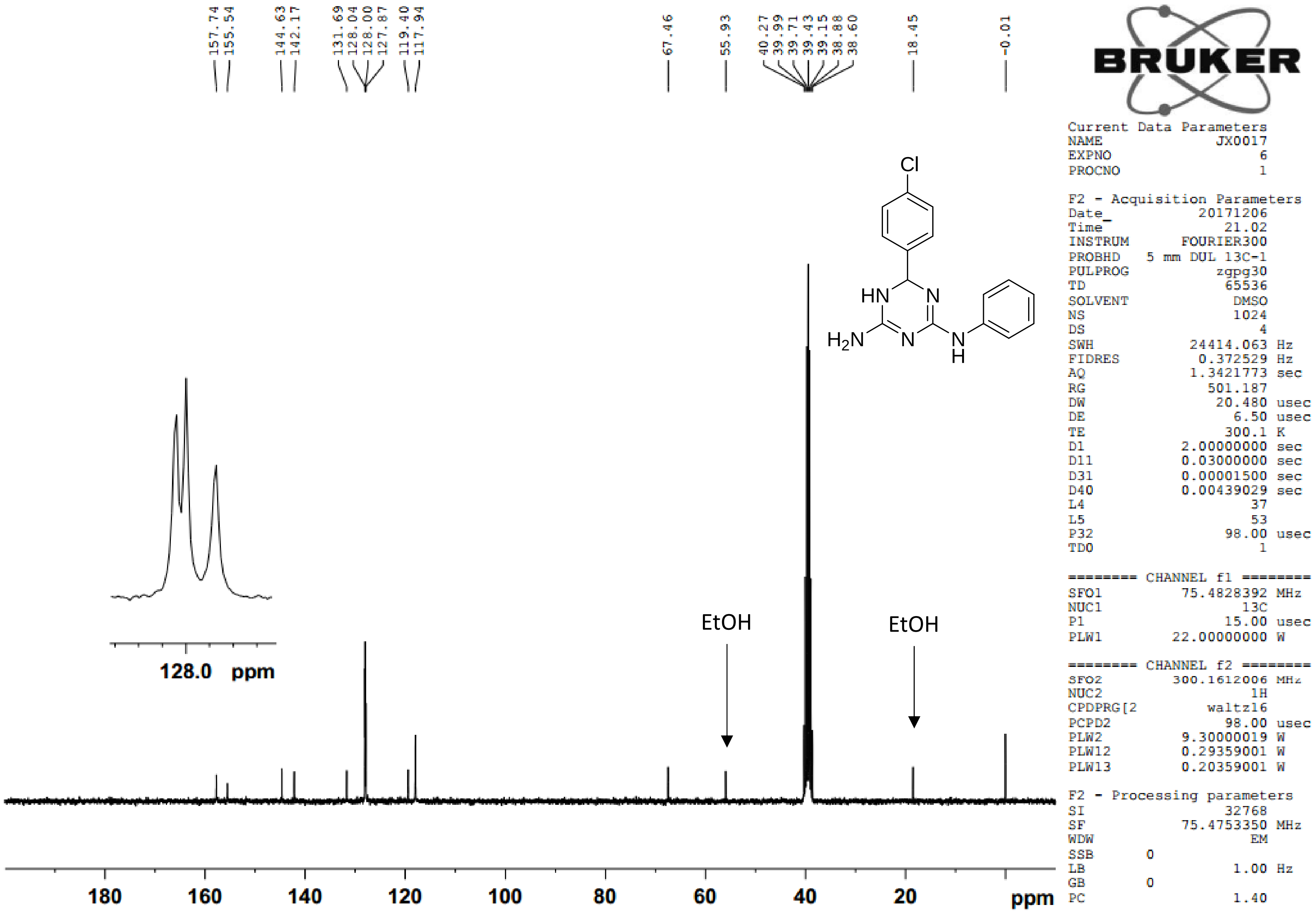

N²,6-Diphenyl-5,6-dihydro-1,3,5-triazine-2,4-diamine (1a)

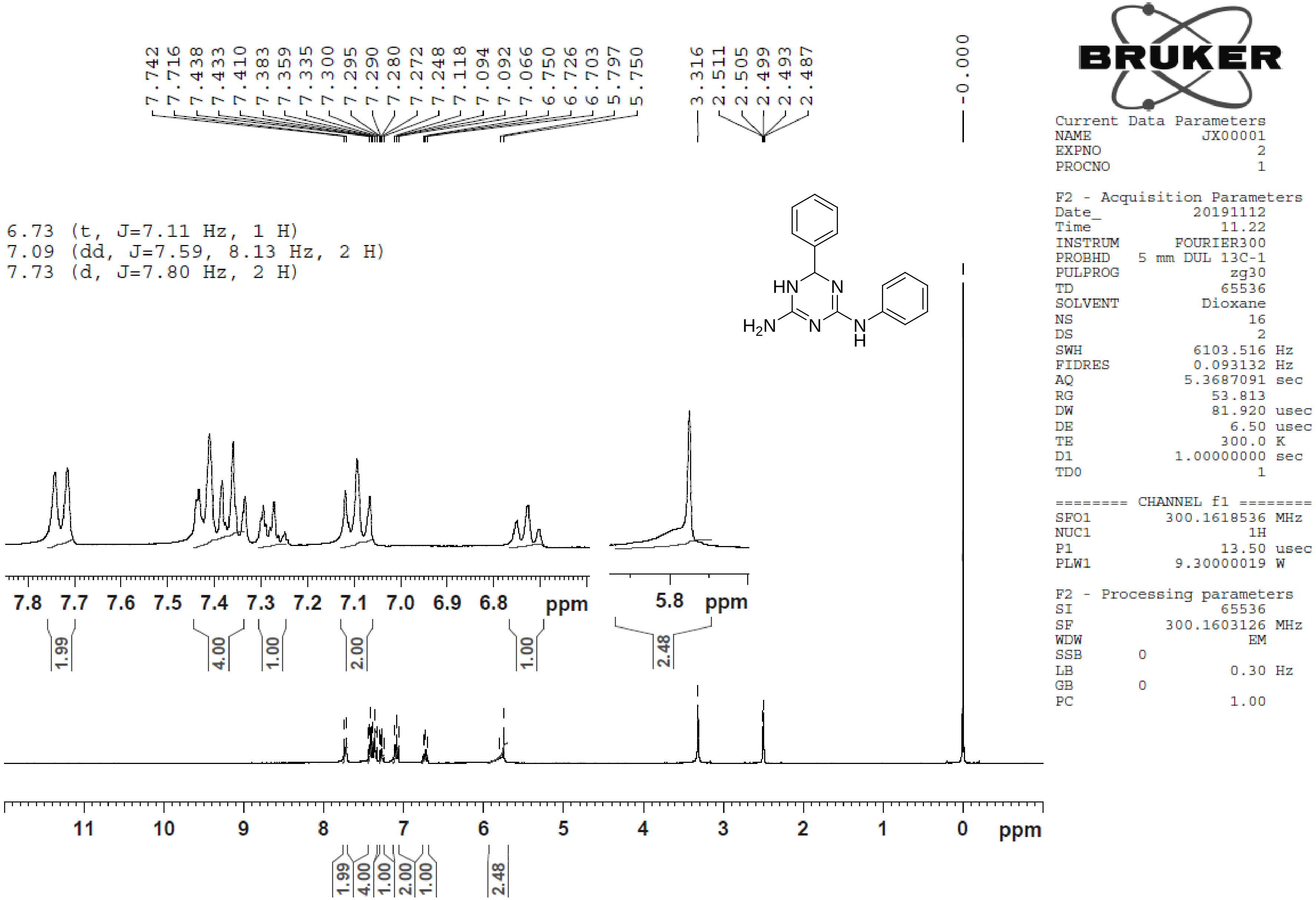

N^2,6-Diphenyl-5,6-dihydro-1,3,5-triazine-2,4-diamine (1a)

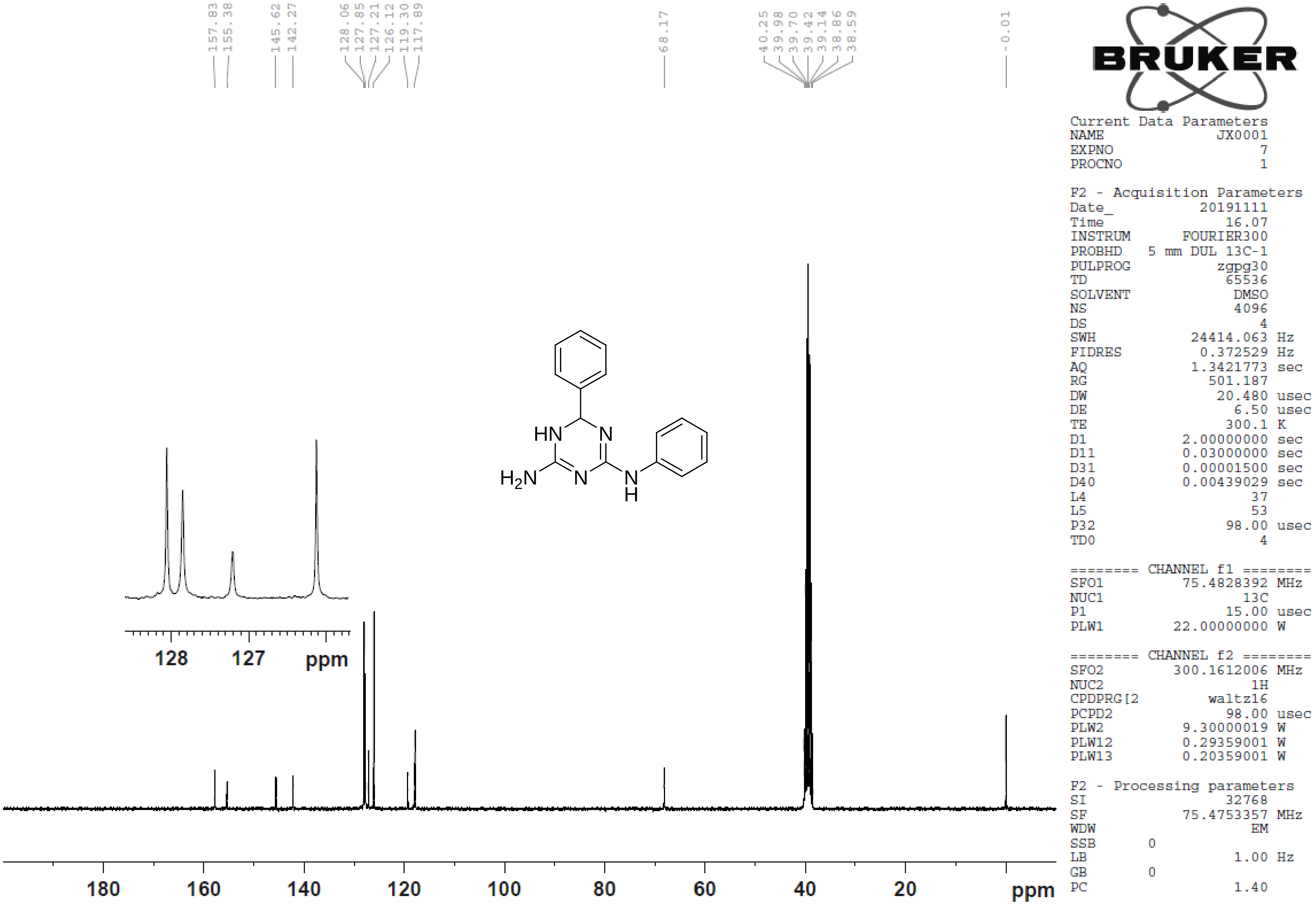

Supplement: RA-009-C9RA08795H-s001 [file RA-009-C9RA08795H-s001.pdf]
